# T-type calcium channels promote predictive homeostasis of input-output relations in thalamocortical neurons of lateral geniculate nucleus

**DOI:** 10.3389/fncom.2014.00098

**Published:** 2014-08-28

**Authors:** Su Z. Hong, Haram R. Kim, Christopher D. Fiorillo

**Affiliations:** Department of Bio and Brain Engineering, Korea Advanced Institute of Science and TechnologyDaejeon, South Korea

**Keywords:** thalamus, retinogeniculate, efficient coding, predictive coding, prediction error, low-threshold spike

## Abstract

A general theory views the function of all neurons as prediction, and one component of this theory is that of “predictive homeostasis” or “prediction error.” It is well established that sensory systems adapt so that neuronal output maintains sensitivity to sensory input, in accord with information theory. Predictive homeostasis applies the same principle at the cellular level, where the challenge is to maintain membrane excitability at the optimal homeostatic level so that spike generation is maximally sensitive to small gradations in synaptic drive. Negative feedback is a hallmark of homeostatic mechanisms, as exemplified by depolarization-activated potassium channels. In contrast, T-type calcium channels exhibit positive feedback that appears at odds with the theory. In thalamocortical neurons of lateral geniculate nucleus (LGN), T-type channels are capable of causing bursts of spikes with an all-or-none character in response to excitation from a hyperpolarized potential. This “burst mode” would partially uncouple visual input from spike output and reduce the information spikes convey about gradations in visual input. However, past observations of T-type-driven bursts may have resulted from unnaturally high membrane excitability. Here we have mimicked within rat brain slices the patterns of synaptic conductance that occur naturally during vision. In support of the theory of predictive homeostasis, we found that T-type channels restored excitability toward its homeostatic level during periods of hyperpolarization. Thus, activation of T-type channels allowed two retinal input spikes to cause one output spike on average, and we observed almost no instances in which output count exceeded input count (a “burst”). T-type calcium channels therefore help to maintain a single optimal mode of transmission rather than creating a second mode. More fundamentally our results support the general theory, which seeks to predict the properties of a neuron's ion channels and synapses given knowledge of natural patterns of synaptic input.

## Introduction

Homeostatic mechanisms are vital to life in general, with numerous examples existing in every cell. Among the most familiar of examples within the nervous system is light adaptation, which allows neurons to maintain sensitivity to small fluctuations in light intensity despite the fact that intensity varies over a large range. Although the value of homeostatic adaptation is obvious, principles from information theory have provided additional insight by providing a means to identify the optimal relationship between input and output, which should correspond to the desired homeostatic target level. The nervous system should counteract (adapt to) patterns in stimulus intensity that are predictable (such as mean intensity) so that neural activity is made to be maximally sensitive to the unpredicted component of stimulus intensity that carries new, “non-redundant” information (Barlow, 1961). Sensory systems have indeed been found to implement efficient (predictive) coding (Laughlin, 1981; Srinivasan et al., 1982; Dan et al., 1996; Rieke et al., 1997; Simoncelli and Olshausen, 2001; Wang et al., 2003; Hosoya et al., 2005; Tobler et al., 2005).

The same principle has been described at the neuronal level as “predictive homeostasis” or “prediction error” (Fiorillo, 2008, 2012; Fiorillo et al., 2014). The homeostatic ideal is for a neuron's spike output to be as sensitive as possible to the amplitude of the excitatory postsynaptic conductance (EPSG) that should cause its spikes. Both the importance and difficulty of achieving this ideal are evident in the fact that synaptic conductance is an analog signal (it can take many values) that varies on a timescale as fast as a millisecond, yet spike output during such a brief period is binary (spike or no spike). Synaptic conductance naturally fluctuates with multiple temporal patterns, and thus a level of membrane excitability that is optimal at one moment could render a neuron's spike output completely insensitive and “blind” to its synaptic input at another moment.

Depolarization-activated potassium channels are well known to promote homeostasis and preserve sensitivity of spike output to input currents (e.g., Wang et al., 2003). By counteracting the depolarization that causes their activation, these channels exhibit the negative feedback that is a hallmark of homeostatic mechanisms. In contrast, positive feedback is notoriously difficult to control. Furthermore, since spikes are caused by synaptic excitation, the need for homeostatic inhibition is more obvious than the need for homeostatic excitation. Since depolarization-activated sodium and calcium channels are excitatory and exhibit positive feedback, they are the antithesis of what one would expect of homeostatic ion channels. One motivation of the present work is to extend the principle of predictive homeostasis to include excitatory ion channels and positive feedback.

In thalamocortical (TC) neurons, T-type calcium channels can exhibit positive feedback powerful enough to cause a “low-threshold spike” (LTS) that is nearly all-or-none and can cause a stereotyped burst of multiple sodium spikes (Llinás and Jahnsen, 1982; Jahnsen and Llinás, 1984; Zhan et al., 1999; Gutierrez et al., 2001). Although evidence eventually emerged suggesting that T-type channels could cause a graded amplification of synaptic excitation under certain conditions (Ulrich and Huguenard, 1997a; Timofeev et al., 2001; Wolfart et al., 2005; Wei et al., 2011; Deleuze et al., 2012), the common belief arose that TC neurons fire in distinct burst and tonic “modes” (Lu et al., 1992; Guido et al., 1995; Reinagel et al., 1999; Sherman, 2001; Lesica and Stanley, 2004; Denning and Reinagel, 2005; Llinás and Steriade, 2006; Wang et al., 2007). At depolarized potentials (~−65 mV), T-type channels are inactivated (Coulter et al., 1989; Crunelli et al., 1989) and TC neurons fire in a “tonic mode” with a nearly linear input-output (I-O) relation. When hyperpolarized (~−80 mV), T-type channels deinactivate and are thereby “primed.” It has been presumed that if synaptic excitation then crosses a threshold, T-type channels will initiate a LTS and subsequent burst of multiple sodium spikes. The I-O relation would therefore be non-linear in the “burst mode,” so that relative to the “tonic mode,” output spike count becomes less sensitive to input spike count and EPSG amplitude.

The role of T-type calcium channels in information processing has been particularly well studied in TC neurons of LGN, which “relay” visual information from retina to cortex. Assuming that the flow of information follows causation, vision in cortex is best served by maintaining the causal link between retinogeniculate EPSGs (rEPSGs) and spike output in TC neurons. However, if T-type channels cause spikes, they would partially uncouple the causal link between retinal input and spike output that is necessary to efficiently transmit visual information. Even if T-type-driven bursts convey visual information and are not truly all-or-none, any reduction in “visual causality” would reduce the information conveyed about gradations in visual stimuli. Although it has been recognized that dual modes of transmission appears problematic, it has been assumed to be an empirical fact, and therefore efforts have been made to understand what advantages might be offered by the burst mode (Guido and Weyand, 1995; Guido et al., 1995; Reinagel et al., 1999; Lesica and Stanley, 2004; Denning and Reinagel, 2005).

If T-type channels drive bursts of spikes in TC neurons, this would contradict the theory of predictive homeostasis, which seeks to generalize the principles of efficient coding and to apply them at the cellular level to understand the function of ion channels. According to this theory, the optimal and homeostatic level of membrane excitability is achieved when a rEPSG depolarizes the membrane precisely to the threshold for the generation of a sodium spike, so that the slightest variation in rEPSG amplitude would determine whether a spike does or does not occur (Fiorillo et al., 2014). A large variety of homeostatic mechanisms are proposed to predict and counteract patterns in rEPSG input (Fiorillo, 2008), resulting in the temporal decorrelation of spike output relative to visual input that has been observed in LGN (Dan et al., 1996). In particular, the theory of predictive homeostasis (Fiorillo et al., 2014) can explain why the average probability that a rEPSG causes a spike is 0.5 (Kaplan et al., 1987; Movshon et al., 2005; Carandini et al., 2007; Sincich et al., 2007; Weyand, 2007; Casti et al., 2008). According to theory, an ion channel subtype performs a homeostatic function if it tends to cause membrane voltage to move toward rather than away from spike threshold under natural conditions. Thus, T-type channels would serve a homeostatic function if they amplify rEPSPs toward spike threshold so that spikes might be generated, but they would oppose homeostasis if their activation consistently causes spikes, as implied by the “burst hypothesis.”

**Figure 1:**
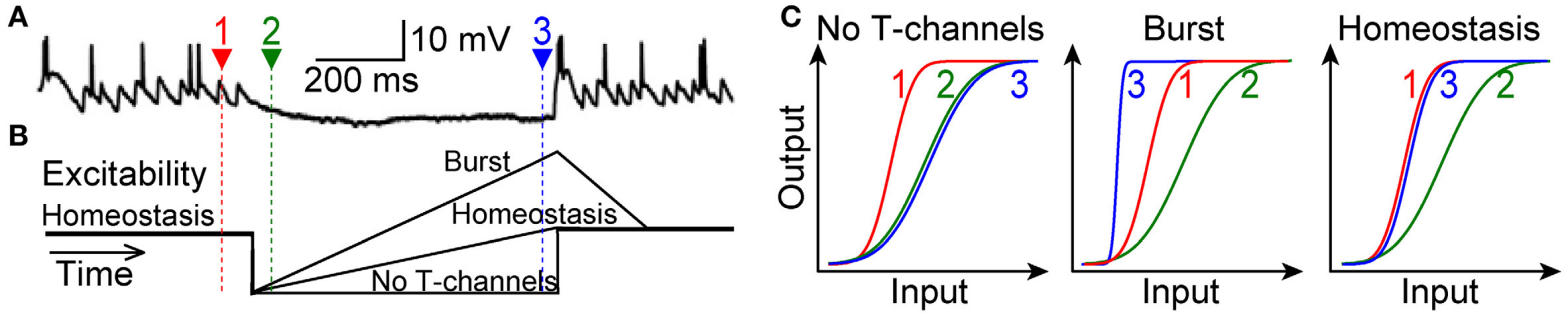
**Three alternative scenarios. (A)** Top, a TC neuron in cat LGN is hyperpolarized for hundreds of milliseconds during viewing of a naturalistic movie (adapted with permission from Wang et al., 2007). Spikes at the end of the hyperpolarization were caused by at least one rEPSG together with T-type current. Actual membrane voltage was not reported. **(B)** Schematic of hypothetical changes in membrane excitability (distance from spike threshold) during hyperpolarization. Homeostatic excitability (1 output spike for 2 input spikes) prevails at depolarized potentials (time 1, red). Hyperpolarization will initially cause a reduction in excitability (time 2, green), but this should recover due to deinactivation of T-type channels. **(C)** Hypothetical I-O functions for retinal input and TC neuron spike output. Relative to the homeostatic and theoretically optimal I-O relation at −65 mV (time 1, red), excitability (at time 3, blue) will either be too weak (left), too strong (middle), or “just right” (right). The burst hypothesis (middle) requires that the I-O relation becomes steeper, but it may or may not shift to the left of the homeostatic I-O relation. In theory, input could correspond to rEPSG amplitude and output to spike probability (Fiorillo et al., 2014), but we measured rEPSG and spike counts and thus I-O relations are not sigmoid (**Figure 8**).

Here we have tested the prediction of theory that T-type channels in LGN do not drive bursts to alter the I-O relation, but rather serve a homeostatic function to maintain the I-O relation. Our primary challenge was to mimic natural visual conditions within rat brain slices. Visually driven transient hyperpolarization partially deinactivates T-type channels, and thus subsequent retinogeniculate excitation will activate them (Figure 1A) (Wang et al., 2007). Our hypothesis was that, in this natural context, activation of T-type channels will amplify retinogeniculate excitatory postsynaptic potentials (rEPSPs) to restore the homeostatic I-O relation of 1 spike for 2 rEPSGs, without causing excess spikes (bursts) (Figure 1B). Our experimental results confirmed this hypothesis. The bursts observed in prior studies were likely the result of the unnaturally high membrane excitability that is typical of brain slice experiments.

## Materials and methods

### Brain slice preparation

Brains were dissected from male Sprague-Dawley rats (21–35 days) anesthetized with CO_2_ as part of a procedure approved by the KAIST Institutional Animal Care and Use Committee. Slices containing dorsal LGN were cut with a vibratome (VT1200, Leica) in a solution (4°C) that contained (in mM): 205 sucrose, 2.5 KCl, 1.2 NaH_2_PO_4_, 7.5 MgCl_2_, 0.5 CaCl_2_, 21.4 NaHCO_3_, 11.1 D-glucose, 1.3 ascorbic acid, 3 pyruvic acid, and 1 kynurenic acid, with pH equilibrated to 7.3 with 95% O_2_ and 5% CO_2_. Slices (200–300 μm) were cut in the coronal plane except in the case of experiments with retinogeniculate EPSGs, in which case slices were parasaggital (400 μm) to better preserve the optic tract, as previously described (Turner and Salt, 1998). Slices were stored in artificial cerebrospinal fluid (ACSF) for at least 30 min prior to being transferred to the recording chamber. During both storage and recording, ACSF was 34°C and had a pH of 7.4 maintained by equilibration with 95% O_2_ and 5% CO_2_. It contained (in mM): 125 NaCl_2_, 2.5 KCl, 1.25 NaH_2_PO_4_, 25 NaHCO_3_, 1 MgCl_2_, 2 CaCl_2_, 10 D-glucose. A slice was submerged in a recording chamber of 0.5 ml and ACSF was perfused at 1.5 ml/min.

### Whole-cell and perforated-patch recordings

Neurons were visualized with gradient contrast infrared optics using an upright microscope (BX51WI, Olympus) with a 60x water-immersion objective. Patch recordings of neurons were performed using a Multiclamp 700B amplifier, Digidata 1320A A/D converter, and pClamp10 software (Molecular Devices, Sunnyvale, CA). Voltage was sampled at 40 kHz and low-pass filtered at 10 kHz. Patch recordings were performed with borosilicate pipettes (3–4 MΩ) filled with a solution containing (in mM): 135 K-methylsulfate, 1.5 MgCl_2_, 0.5 EGTA, 10 HEPES, 2 Mg-ATP, 0.2 Na_2_-GTP, and 10 phosphocreatine, with pH adjusted to 7.3 with KOH (osmolarity 280 mOsm). Pipette solution was the same for perforated-patch recordings, except for the inclusion of 50 μM β-escin. Sufficient membrane perforation required 20–30 min, with final series resistances ranging from 10 to 20 MΩ. In all recordings, access and series resistance was monitored periodically using the “membrane test” function of pClamp10. Data was discarded if series resistance exceeded 25 MΩ, or if there was a change of greater than 20% from its initial value. Correction was made for a liquid junction potential of +10 mV.

The pipette's chloride concentration was lower than in typical whole-cell solutions to maintain the natural chloride equilibrium potential. We confirmed, through experiments with iontophoresis of GABA, that the chloride reversal potential was near its reported level of −80 mV in TC neurons (Ulrich and Huguenard, 1997b). The low chloride internal solution was designed for experiments in which membrane excitability was to be measured in the presence of GABA. However, data from such experiments is not presented here, and thus the chloride equilibrium potential was presumably irrelevant for interpretation of the reported data.

### Identification of TC neurons in dorsal LGN

Most of the boundary of dLGN was easily distinguished in living tissue, both in the coronal plane as well as the parasagittal plane used for retinogeniculate EPSPs. The ventromedial boundary (in coronal slices) was more difficult to see, although the oval shape of dLGN allowed its position to be inferred. To be conservative, recordings were made at least 100 μm from the inferred ventromedial boundary. Most recordings were near the center of dLGN, ~4–5 mm caudal of Bregma.

TC neurons in dLGN were distinguished from interneurons by established criteria. Prior to patching, a neuron was selected that had more than two dendrites extending from the soma. TC neurons were subsequently distinguished from interneurons by the presence of a rebound LTS with a burst of multiple sodium spikes in response to sudden offset of a large hyperpolarizaing current (indicative of T-type current), a depolarizing “sag” following strong hyperpolarization (indicative of H-type current), and resting membrane resistances not greater than 300 MΩ. Cells judged to be TC neurons had resting potentials of −71.4 ± 6.2 mV and membrane resistances at resting potential of 180 ± 43 MΩ (mean ± SD, *n* = 87).

### Retinogeniculate EPSPs

Retinogeniculate EPSPs were evoked by electrical stimulation (16–30 μA, 0.2 ms) using a concentric bipolar stainless steel electrode placed at the point at which the optic tract enters thalamus (FHC Inc., Bowdoin, ME). Experiments were performed in the presence of the GABA_A_ receptor antagonist picrotoxin (100 μM). In addition to the site of stimulation being within the optic tract, excitatory postsynaptic currents (EPSCs) were judged to be of retinal origin (rather than cortical) by their large unitary amplitude and strong paired-pulse depression (Turner and Salt, 1998; Chen et al., 2002). In each of the 9 neurons (from which rEPSP data is reported), EPSCs were recorded in neurons voltage-clamped at −70 mV. The amplitude of extracellular stimulation current was increased in small increments (2 μA), and a rEPSC was distinguished by a large and abrupt increase in EPSC amplitude (>280 pA) that did not increase substantially with additional current. In 3 of the 9 neurons for which data is reported, we observed two large increases in amplitude, consistent with the known existence of up to several retinogeniculate inputs in some TC neurons. Neurons were excluded from analysis if no large and abrupt increase in EPSC amplitude was observed. EPSCs were subsequently evoked in pairs (with an interval of 50 ms) every 20 s, and were observed to exhibit strong depression (the amplitude of the second EPSC being 70% or less of the first EPSC in all 9 neurons). EPSCs were blocked by the AMPA-receptor antagonist CNQX (10 μM).

We wished to mimic the natural condition *in vivo* in which temporal summation of 2 EPSGs is necessary to cause 1 spike. However, in many cells we found that a spike occurred in response to the first rEPSG evoked from −65 mV, and that additional rEPSGs caused a very small increase in rEPSP amplitude and did not result in additional spikes. This discrepancy from the *in vivo* case is likely to be because the retinogeniculate synapse is in a “depressed state” under natural conditions *in vivo*, and therefore “paired pulse” interactions are small and rEPSG amplitude varies little over time (Borst, 2010). There are several factors that probably act to reduce paired-pulse interactions *in vivo*, including moderately high firing rates in retinal ganglion neurons and lower extracellular calcium than the 2.0 mM used here and in most studies *in vitro* (Borst, 2010). Another factor is likely to be suppression of vesicle release by presynaptic G-protein coupled receptors, which can virtually eliminate paired-pulse depression (Brenowitz and Trussell, 2001). We therefore performed experiments in a low concentration of the GABA_B_ receptor agonist baclofen (0.05–1.0 μM across the 9 cells), which reduced paired-pulse depression of rEPSCs evoked 50 ms apart from a control level of 0.58 ± 0.12 to 0.86 ± 0.20 in baclofen (mean ± SD of second to first rEPSC amplitude, *n* = 9). In the presence of baclofen, temporal summation of EPSPs was more pronounced, so that two rEPSGs were often necessary and sufficient to cause one spike.

For most aEPSG events and all rEPSG events, an interval of 5.0 ms was used between single EPSGs. This is at least twice the absolute refractory period for spikes in retinal ganglion neurons *in vivo*. However, the absolute refractory period in our brain slices could have been longer, at least in part because of our lower temperature (34°). This could have resulted in failure of some of our electrical stimuli to evoke presynaptic spikes in retinal axons (complete failures of vesicle release given a spike seem unlikely, since the retinogeniculate synapse is a large synapse with many release sites, and our use of baclofen suppressed paired-pulse depression). We therefore visually inspected rEPSPs to look for transmission failures. We focused on rEPSPs evoked after 50 ms at −80 mV (since these did not result in spikes), especially the case of events with 4 rEPSGs. Successful transmission could be recognized as positive slope about 3–5 ms after each electrical stimulus (interrupted at 5 ms in some cases by the electrical artifact of the subsequent stimulus), and a subsequent lack of substantial decay of the rEPSP after about 7–10 ms (**Figure 4A**). In only 1 of the 9 cells did we observe failures, and these always occurred for the 3rd of 4 rEPSGs (for 8 of 8 rEPSPs after 50 and 100 ms at −80 mV). Nonetheless, this cell displayed a typical profile in terms of its I-O counts. In particular, it had more spikes in response to 4 rESPGs than 2 rEPSGs, both at −65 mV (1.9 vs. 0.9) and after 400 and 800 ms at −80 mV (1.4 vs. 0.9). Thus, presynaptic spike failures appeared to be infrequent and relatively inconsequential. If such failures had made a substantial contribution, they would not necessarily have altered the relative excitability at −80 vs. −65 mV. Indeed, even when 4 aEPSGs with no paired-pulse depression were delivered with 2.0 ms intervals (500 Hz), excitability at −80 mV did not exceed that at −65 mV (**Figure 6**).

### Dynamic clamp

Dynamic clamp was used to deliver artificial synaptic conductances. It utilized an Intel Quad Core computer equipped with an acquisition card (NI PCI-6251 ADC/DAC, M series, National Instruments, Austin, TX) connected to a Multiclamp 700B amplifier, operating in current-clamp mode, via a Digidata 1320A A/D converter (Molecular Devices, Sunnyvale, CA). Artificial conductance was generated by Windows-based dynamic clamp software, StdpC2012 (Nowotny et al., 2006; Kemenes et al., 2011).

### Natural patterns of synaptic conductances

Our artificial conductances should mimic the sum of all synaptic conductances that are typically present during a rEPSP. In addition to the rEPSGs and opponent-type IPSGs described in Results, synaptic inhibition from local interneurons with the “same sign” (e.g., an ON-type interneuron inhibits an ON-type thalamocortical neuron) typically has an onset about 1 ms after rEPSG onset and could therefore have a homeostatic role in suppressing rEPSPs (Blitz and Regehr, 2005; Lindström and Wróbel, 2011; Wang et al., 2011; Vigeland et al., 2013). There is also a relatively tonic inhibition mediated by high-affinity, extrasynaptic GABA_A_ receptors that is probably greater *in vivo* than *in vitro* (Cope et al., 2005; Bright et al., 2007). There are corticogeniculate EPSGs on distal dendrites, but their timing with respect to rEPSGs is not known. They are believed to be “modulatory” in the sense that although they depolarize the membrane, unlike rEPSGs they are not the proximal cause of spikes (Sherman and Guillery, 1998; Fiorillo et al., 2014).

Retinogeniculate excitation and opponent inhibition will naturally be anti-correlated with one another (since it is rare to have simultaneous evidence both for and against light in the receptive field center) (Mastronarde, 1989). Therefore, periods of hyperpolarization will usually be accompanied by an absence of rEPSGs, and the onset of a rEPSG will often coincide with the offset of opponent inhibition, as in the example of Figure 1A. However, homeostatic, “same-sign” synaptic inhibition presumably tends to accompany rEPSGs regardless of whether the rEPSGs occur at depolarized or hyperpolarized potentials. Therefore, synaptic inhibition is likely to be at a high level throughout the period in which T-type channels are activated. Our artificial conductances should ideally mimic the dynamically varying sum of both types of synaptic inhibition as well as rEPSGs. However, for simplicity, we did not attempt to mimic homeostatic synaptic inhibition during EPSG events, at either −65 or −80 mV.

### Artificial EPSGs

The waveform of an aEPSG was delivered via dynamic clamp (see above) and was based on “Destexhe Synapses” (Destexhe et al., 1994) built in StdpC2012 with the following parameter values for maximum conductance (gSyn = 7–12 nS), reversal potential (Vrev = 0 mV), duration of extracellular neurotransmitter (tRelease = 1.0 ms), forward rate constant (alpha = 0.11/ms), and backward rate constant (beta = 0.19/ms). Our aEPSGs were designed to mimic only the AMPA-receptor-mediated component of rEPSGs, and not the more complex NMDA-receptor component.

Although paired-pulse depression is likely to occur *in vivo*, its effect is modest (see Retinogeniculate EPSPs). Thus, each of our aEPSGs was of equal amplitude, both within and across events within a single neuron. With our standard 5 ms inter-EPSG intervals, our aEPSPs displayed less PPD than our rEPSPs (**Figures 4A,B**), but they were more effective in reproducing the natural I-O relation. Relative to natural conditions, we suspect that our aEPSGs had too little PPD and that our rEPSGs had too much (possibly related to our use of 2.0 mM external calcium; Borst, 2010).

All experiments with aEPSGs began by adjusting aEPSG amplitude so that the second but not the first of 2 aEPSGs would cause 1 spike from a potential of −65 mV. During this initial adjustment process, events consisted of 1 aEPSG in isolation, and in alternating sweeps, a pair of 2 aEPSGs separated by 5.0 ms (or 2 or 10 ms in 7 cells), each individual aEPSG having an equivalent peak amplitude. The amplitude of aEPSGs was gradually increased until one spike was evoked by the pair of aEPSGs but not by the single aEPSGs. The amplitude was then further increased until the single aEPSG was sufficient to evoke a spike (which was always observed to occur with a lesser aEPSG amplitude than that required to evoke 2 spikes with 2 aEPSGs). We then selected as our standard peak aEPSG amplitude (for that cell) the average of the lower value (for which only the 2nd aEPSG of the pair caused a spike) and the higher value (for which just a single aEPSG caused a spike). This resulted in a range of peak amplitudes across cells of 7 −12 nS.

Peak aEPSG amplitudes (7–12 nS) were similar to those estimated for rEPSGs (6–18 nS based on voltage-clamp recordings). Likewise, aEPSPs were virtually identical to rEPSPs in amplitude (15.7 ± 3.3 for aEPSPs vs. 15.2 ± 3.6 mV for rEPSPs at −80 mV, mean ± SD) and half-decay time (20.0 ± 6.2 for aEPSPs vs. 24.9 ± 10.4 mV for rEPSPs at −65 mV, 12.5 ± 2.8 for aEPSPs vs. 13.1 ± 3.1 for rEPSPs at −80 mV) in response to one EPSG. Since our aEPSG waveform was not designed to mimic the NMDA-receptor component of the EPSP, this might account for the very slight tendency of our aEPSPs to decay faster than rEPSPs, especially for EPSPs evoked from −65 mV. The substantially faster decay of both aEPSPs and rEPSPs at −80 mV relative to −65 mV was presumably due to a faster membrane time constant as a result of the higher membrane conductance at −80 mV.

### Artificial inhibition

Artificial inhibitory conductance (3–10 nS) was applied for 1.0–1.2 s with a square wave pattern. Its reversal potential was adjusted in each cell to cause hyperpolarization to near −80 mV, which required reversal potentials between −85 and −100 mV across cells. These values are between the measured equilibrium potentials in TC neurons for chloride (−80 mV) (Ulrich and Huguenard, 1997b) and potassium (−100 to −110 mV). Although our intention was to mimic synaptic inhibition, we did not attempt to mimic the waveform of unitary inhibitory postsynaptic conductances because we wanted to study EPSPs and TTDs without the complexity added by dynamic inhibition. Furthermore, the waveform of unitary IPSPs is not visible during synaptically-mediated hyperpolarization of thalamocortical neurons in LGN (Figure 1A) (Wang et al., 2007, 2010).

In experiments with square-wave depolarizing currents, hyperpolarization to near −80 mV was achieved by injection of an artificial conductance of 10 nS for 1.2 s. This same conductance (10 nS) was previously shown to suppress generation of the LTS through shunting (Ulrich and Huguenard, 1997a). It should ideally mimic the summed conductance of both the synaptic inhibition that causes hyperpolarization as well as the synaptic excitation that activates T-type channels. Resistance at resting potentials was 152 ± 55 MΩ (mean ± SD, range 90–300 MΩ at −70 ± 4 mV in 22 neurons), and the artificial conductance would be expected to reduce this to 59 ± 8 MΩ (47–75 MΩ) if we disregard the fact that membrane resistance would be somewhat lower at −80 due to an increase in H-type conductance. In comparison, the average resting *in vivo* membrane resistance of TC neurons under anesthesia was estimated to be 22 MΩ with sharp electrodes (Vigeland et al., 2013) and less than 65 MΩ (the series resistance) with patch electrodes (Margrie et al., 2002).

In experiments with rEPSGs and aEPSGs, the membrane was hyperpolarized to near −80 mV (1.0 s) by an artificial conductance (3–10 nS, mean 6 nS) that was equal to the resting conductance of the cell and thus would be expected to reduce membrane resistance by half from its resting level at −65 mV of 191 ± 43. Previous efforts to mimic *in vivo* conditions by application of artificial conductances also reduced membrane resistance by about one half (Wolfart et al., 2005; Deleuze et al., 2012), or more (Ulrich and Huguenard, 1997a) (this excludes the conductance of the real or artificial retinogeniculate EPSG, both here and in previous studies). Our artificial conductances may be somewhat low in comparison to natural synaptic conductance. A single pulse of extracellular electrical stimulation caused a 75% reduction of membrane resistance at the peak of a GABA_A_ IPSG in TC neurons in brain slices (Crunelli et al., 1988).

### Estimates of membrane conductance in our experiments

At the peak of single rEPSGs and aEPSGs (mean = 9.5 nS), membrane conductance increased 2.7-fold above its mean resting level (near −70 mV) of 5.5 nS. At the peak of the 4th aEPSG in an event, the total aEPSG conductance was about 15 nS (1.6-fold greater than the peak of a unitary aEPSG, as a result of temporal summation). When we consider the additional artificial conductance that caused hyperpolarization and was intended to mimic opponent-type synaptic inhibition (mean of 5.5 nS), the total artificial conductance at the peak of a single aEPSG would be about 15 nS, and total membrane conductance would reach an average peak greater than 21 nS (it would be greater considering that the estimate of 21 nS neglects the increased in conductance due to opening of H-type cation channels following hyperpolarization to −80 mV). In comparison, Vigeland et al. (2013) performed *in vivo* intracellular recordings in LGN and reported that rEPSPs caused total membrane conductance to increase to about 86 nS from a resting level of 46 nS. This estimate was based on average synaptic conductance over a 20 ms period, which included simultaneous rEPSGs and homeostatic (“same-sign”) synaptic inhibition, but did not include opponent-type synaptic inhibition. The lower conductances in our cells were likely due at least in part to the loss of some dendrites during slice preparation.

### Temporal aspects of experimental designs

**Figure 2:**
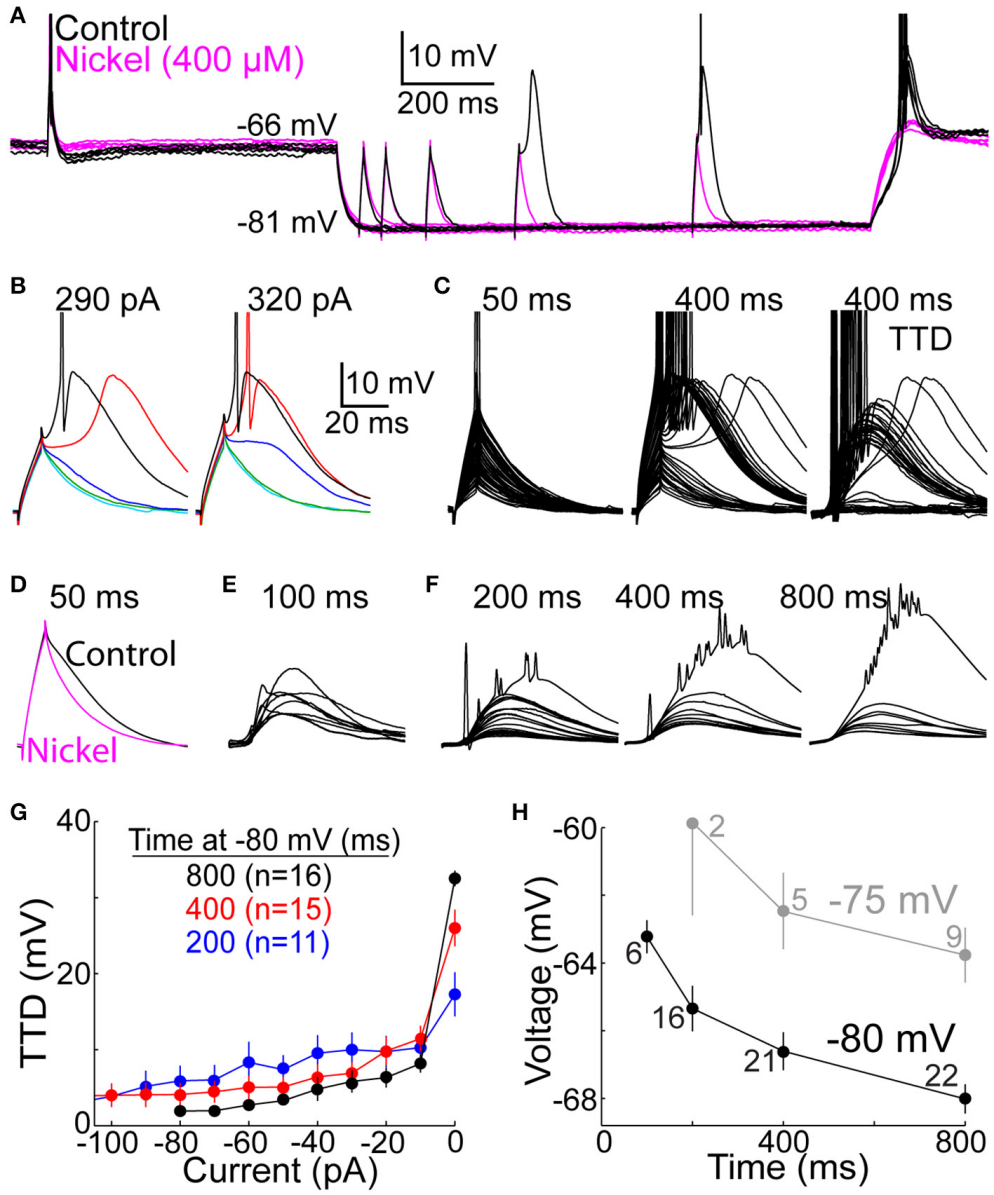
**The T-type depolarization has a graded character. (A)** Overlay of membrane voltage during 10 sweeps. On each sweep, membrane potential was near −65 mV (for 2.0 s) before and after hyperpolarization to near −80 mV by injection of an artificial conductance (10 nS for 1.2 s). Depolarizing currents were injected for 10 ms at −65 mV, and after a delay following hyperpolarization of 50, 100, 200, 400, or 800 ms (the illustrated current was 290 pA). The depolarization increased over time at −80 mV under control conditions (black) but not in the presence of nickel (400 μM) (magenta). **(B)** Voltage responses to currents of 290 (left) and 320 pA (right) at 50 (cyan), 100 (green), 200 (blue), 400 (red), and 800 ms (black) after onset of hyperpolarization in the same cell shown in **(A)**. Scale bars apply to **(B–F)**. **(C)** Voltage responses to all current increments at 50 (left) and 400 ms (middle) in the same cell as **(A,B)**. At right, voltages at 50 ms were subtracted from those at 400 ms to isolate the TTD (albeit with sodium spikes). **(D)** The decay of depolarization at 50 ms was accelerated in the presence of nickel (400 μM) (magenta), indicating a small TTD. Each of the two traces represents the average response across 5 cells to the largest current that did not evoke a sodium spike. **(E)** The TTD at 100 ms in 9 cells (other cells included in **(F)** were not tested at 100 ms) in response to the largest current in each cell which did not cause a sodium spike (430–650 pA). **(F)** Average TTDs at 200, 400, and 800 ms. For each cell, we identified the smallest current increment that caused at least one sodium spike, and we averaged the voltage response to that current and all smaller currents (−10 pA, −20 pA, etc.) across all cells. The largest TTD at each time shows the remnants of sodium spikes that appear diminished in amplitude due to averaging. **(G)** Following the method in **(F)**, the amplitude of the TTD required to cause a spike (“0 pA”) and all smaller TTDs is plotted as a function of current for 200 (blue), 400 (red), and 800 ms (black). Fewer cells contributed data at shorter delays here and in **(F)**, since no spikes were evoked by the largest tested current in some cells. **(H)** LTS thresholds were measured as the peak voltage reached at the end of the largest current injection that did not cause a LTS. Each point represents the mean (±s.e.m.) across 2–22 neurons, as indicated. The experimental design used to evoke TTDs from −75 mV (gray) was identical to that at −80 (black), including application of an artificial conductance of 10 nS.

Our standard protocol consisted of alternating periods of a depolarized state near −65 mV (2.0 s) and a hyperpolarized state near −80 mV (1.0–1.2 s) (Figures 2A, **4A,B**). The depolarized potential was adjusted to ~−65 mV through current injection. The hyperpolarization was achieved by injection of an artificial conductance (except current alone in the experiments of **Figure 10**) with a square-wave pattern.

**Figure 3:**
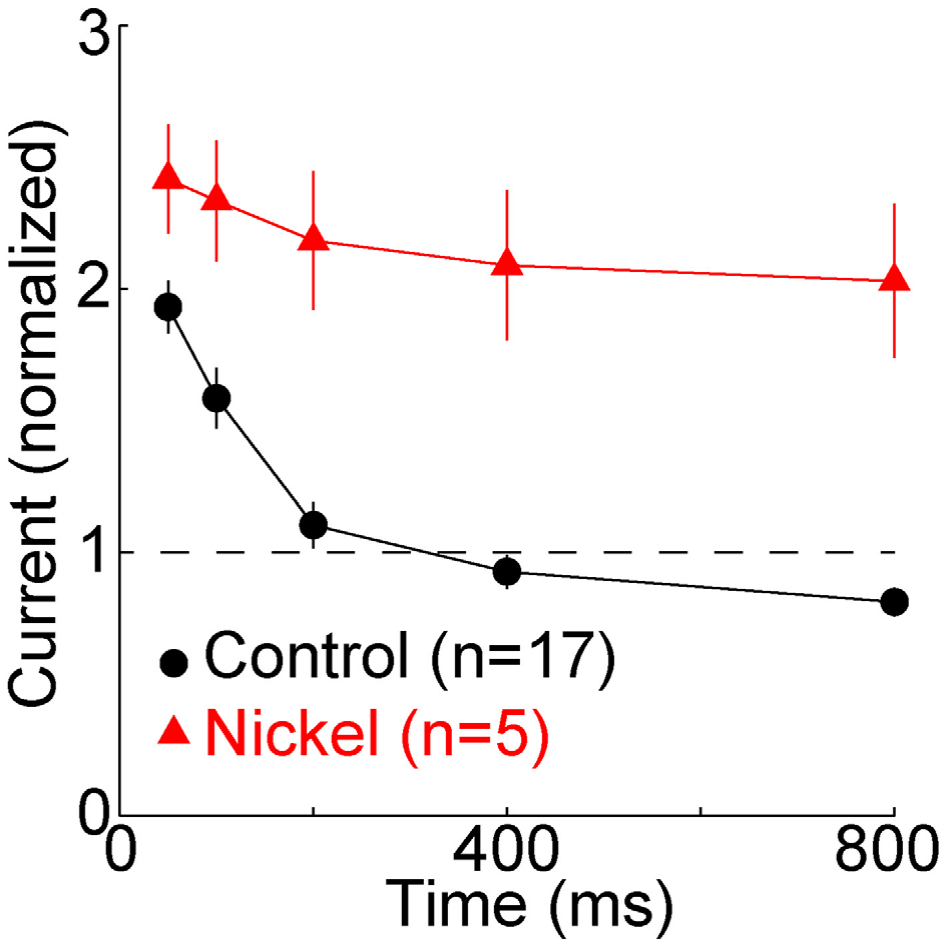
**T-type calcium channels restore membrane excitability**. The minimal “threshold” current (mean ± s.e.m., *n* = 17, black) necessary to evoke one or more sodium spikes from −80 mV was divisively normalized by the threshold current at −65 mV (432 ± 128 pA, mean ± SD). In a subset of these cells (*n* = 5, red), nickel (400 μM) was subsequently perfused. It typically caused a depolarization of a few mV. After readjusting holding current and the reversal potential of the artificial conductance to restore membrane potentials to ~-65 and −80 mV, the same protocol was repeated. The threshold currents in nickel were normalized to the threshold current from −65 mV in nickel (333 ± 65 pA), which was less than in control conditions (432 ± 128 pA).

In experiments with square-wave currents, depolarizing currents of 10 ms duration were injected from −65 mV, or from −80 mV after delays of 50, 100, 200, 400, and 800 ms following onset of hyperpolarization. Currents were increased over successive sweeps in increments of 10 pA over a range of 200–900 pA. However, in a subset of 7 cells tested with two concentrations of external calcium, the range was 100–1000 pA. These cells contributed to Figures 3, **12**. In another subset of 5 cells, 10 pA increments were used from 200 to 500 pA, and 50 pA increments between 500 and 900 pA. This latter subset was therefore excluded in Figures 2F,G. In all cells, one current increment was tested during each 3.0 s “sweep,” starting with the smallest current. During a single sweep, a current was injected at −65 mV (at 650 ms prior to hyperpolarization) (Figure 2A), and a current of the same amplitude was then injected from −80 mV. All current increments were tested at 50 ms before proceeding to longer delays, ending with 800 ms. A single increment size at a single delay was delivered once per cell. Currents were injected at delays of 100 ms in only 9 of the 22 neurons for which data is reported.

**Figure 4:**
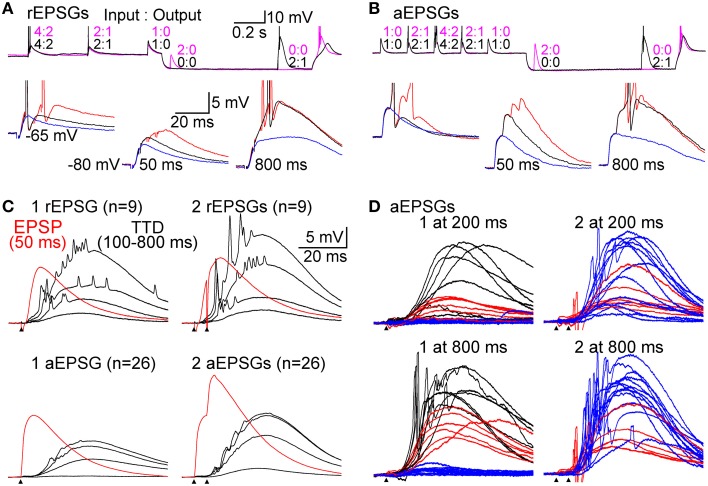
**Graded amplification of EPSPs**. Events consisting of 1, 2, or 4 unitary EPSGs separated by 5 ms intervals were evoked at −65 mV and after delays of 50–800 ms after injection of an artificial conductance (3–10 nS for 1.0 s) that hyperpolarized the membrane to near −80 mV. **(A)** Top, two separate sweeps in which 2 rEPSGs occurred either at 50 (magenta) or 800 ms (black). Numbers indicate EPSG input count and spike output for each EPSG event. Bottom, overlaid rEPSPs in response to 1 (blue), 2 (black), and 4 (red) rEPSGs at −65 mV (left), and after 50 (middle) and 800 ms (right) at −80 mV. Scale bars also apply to **(B)**. **(B)** Same as in **(A)**, but for aEPSGs from another cell. **(C)** Population mean TTDs at 100, 200, 400, and 800 ms in response to 1 (left) and 2 (right) EPSGs. rEPSGs at top, aEPSGs at bottom. Population mean EPSPs at 50 ms (red) are overlaid for comparison. Scale bars apply to all traces in **(C)** and **(D)**. **(D)** Temporal summation illustrated by TTDs in response to 1 (left) and 2 (right) aEPSGs at 200 (top) and 800 ms (bottom). Each trace is an average of 4 repetitions in one cell. The traces from each cell were assigned a color based on the response of that cell to 1 aEPSG at 800 ms (lower left), according to whether there was virtually no TTD (blue; *n* = 13), a large TTD that caused no spike (red; *n* = 6), or a TTD that caused one or more spikes (black; *n* = 7). Each cell is represented by the same color in the other three conditions, but to aid clarity, cells with spikes in response to 1 aEPSG at 800 ms are not shown in the case of 2 aEPSGs. If little TTD was present in response to 1 aEPSG, the second aEPSG caused a large TTD. In other cells with a large TTD but no spikes, the second aEPSG caused at least one spike. Similar results were obtained with rEPSGs.

In experiments with EPSGs, multiple EPSG events were delivered during each 2.0 s period at −65 mV, but just one event was delivered at −80 mV (Figures 4A,B). 5 aEPSG events were delivered in each of two consecutive seconds at −65 mV (10 events total), with a pattern of 1, 2, 4, 2, 1 unitary aEPSGs per event (20 total aEPSGs) (Figure 4B). The same design was used for 2 of the 9 cells for which data is reported with rEPSGs, but half of that rate was used for the other 7 cells (5 total events were spread over 2.0 s, with 1, 2, 4, 2, and 1 rEPSGs per event). This allowed us to measure excitability from a typical depolarized potential, and to roughly mimic naturally occurring levels of EPSG input and spike output (about 14 and 7 Hz, respectively). The depolarized potential may also have kept T-type channels in their normal phosphorylated and “potentiated” state (Bessaïh et al., 2008).

One EPSG event was delivered during each 1.0 s period at −80 mV, following delays from hyperpolarization onset of 50, 100, 200, 400, and 800 ms. Each of the 3 events (1, 2, and 4 EPSGs) was tested at each of the 5 delays in 15 consecutive “sweeps” within a single “block,” starting with 1 EPSG and progressing to 4 EPSGs. Within each block, delays were tested in the order 50, 800, 100, 400, and 200 ms. The block was then repeated 4 times, for a total of 4 repetitions of each condition (EPSG count and delay) at −80 mV. In the case of rEPSGs, all the data reported were from blocks 2 to 4. The first block was excluded in order to allow time for paired-pulse interactions and rEPSP amplitudes to reach a steady state.

### Data analysis

Data were analyzed using Matlab (Natick, MA). TTD amplitude in Figure 2G was measured after smoothing with a 10 ms moving average (incremented by steps of 1 ms) in order to minimize the contribution of sodium spikes. The peak of the TTD was considered to be the maximum value of the smoothed trace, measured from baseline voltage prior to current injection (near −80 mV).

For analysis of the effect of lower calcium on TTD amplitude, we pooled across conditions in order to maximize statistical power to assess what was a modest effect. For each cell, one average TTD was selected in 2.0 mM calcium and another in 1.2 mM calcium. To find this one average TTD for each cell, we identified the largest TTD for each of the four time points (100, 200, 400, and 800 ms) from among all TTDs in response to each input condition (current amplitude in our square-wave protocol, or aEPSG count). We excluded any responses with one or more sodium spikes. We then averaged across these four TTDs in each cell to find the average TTD for that cell and calcium concentration. Although the input conditions (current or aEPSG count) varied across cells, the input was identical for both calcium concentrations in any given cell. We then made paired comparisons of TTD amplitude across the population of 17 cells (7 in the square-wave protocol and 10 in the aEPSG protocol) (**Figure 12C**).

Sodium-based action potential thresholds were quantified in each of the two calcium concentrations by visual inspection. The viewer was “blind” to the calcium concentration. This was done in 17 cells, 7 in the square-wave protocol and 10 in the aEPSG protocol. For the 7 cells recorded with the square-wave protocol, we also estimated action potential thresholds based on the peak voltage achieved in response to the largest current injection that did not cause a spike. Because current-increments were small (10 pA for 10 ms), voltage increments were also small (~0.2 mV), and thus this was a sensitive technique. Confirming the lower thresholds found in all 17 cells based on visual inspection (see Calcium Concentration in Results), spike thresholds measured in this way were also significantly lower in 1.2 mM (−54 ± 3.5 mV, mean ± SD) vs. 2.0 mM calcium (−50 ± 2.0 mV) (*n* = 7, *p* = 0.003, paired *t*-test).

### Chemicals

All chemicals were obtained from Sigma (St. Louis, MO), except potassium methylsulfate from Acros Organics (Geel, Belgium).

### Comparison to previous efforts to mimic natural physiological conditions of T-type channel activation

Like the present study, both Wolfart et al. (2005) and Deleuze et al. (2012) studied T-type calcium channels in TC neurons while using dynamic clamp to mimic aspects of natural synaptic input. Although not motivated by the theoretical issues explored here, the latter study in somatosensory (ventrobasal) thalamus also found that T-type calcium channels maintain I-O relations during hyperpolarization (over the range of −55 to −72 mV). Their results are fully consistent with ours and support the theory of predictive homeostasis. Here we describe several respects in which our experiments are distinct from these two prior studies and may be more relevant to natural physiological conditions.

First, both prior studies examined the case of sustained hyperpolarization (lasting at least seconds), whereas we examined responses during mimicry of the transient (sub-second) periods of visually driven hyperpolarization observed *in vivo*, which cause only partial deinactivation of T-type channels. Second, whereas the input variable of their I-O relation (and that of Wolfart et al., 2005) was the amplitude of unitary aEPSGs, unitary rEPSG amplitude has not been found to vary substantially *in vivo* (Carandini et al., 2007; Sincich et al., 2007; Borst, 2010), as suggested by inspection of Figure 1A (Wang et al., 2007). The more critical input variable is the number of temporally summed rEPSGs (Carandini et al., 2007; Sincich et al., 2007; Weyand, 2007; Casti et al., 2008), which we mimicked (Figure 4).

A third distinction is that both prior studies applied a random background “noise” conductance (via dynamic clamp). This variable conductance was intended to mimic the effect of corticogeniculate activity, and it was shown by Wolfart et al. (2005) to have a powerful effect in flattening the I-O relation (after averaging across trials with identical artificial retinogeniculate “input” conductance but variable “noise” conductance). Although corticogeniculate activity presumably has an important modulatory function on the retinogeniculate I-O relation (Sherman and Guillery, 1998), the timing of corticogeniculate activity relative to retinogeniculate input is unknown. We presume that it is neither random nor contributes noise under conditions of active vision. In addition, the level of membrane potential variance added via dynamic clamp (*SD* = 3.6 mV in the case of Wolfart et al., 2005, and presumably comparable in the study of Deleuze et al., 2012) appears to have been considerably greater than that observed during visually-driven hyperpolarization of thalamocortical neurons recorded *in vivo* (~1 mV or less in anesthetized animals) (Figure 1A) (Wang et al., 2007, 2010). The sub-millivolt variance in our experiments may therefore have been closer to the *in vivo* case.

Fourth, we went beyond both of these prior studies insofar as we verified our central observations with real retinogeniculate EPSPs, with perforated-patch pipettes (in combination with aEPSGs) to maintain the natural cytosolic constituents of neurons, and with a lower and more physiological concentration of extracellular calcium.

## Results

To understand the experimental design and judge its ability to address the hypothesis, it is first necessary to have knowledge of both the homeostatic I-O relation and natural conditions in TC neurons. We therefore address these issues in relation to our specific hypothesis before we present experimental results. We often refer to synaptic conductances (EPSGs) rather than potentials (EPSPs) because they are the input to neurons, they are relatively independent of membrane properties, and they are more directly under our control.

### Natural conditions of T-type channel activation

LGN was chosen for this study in part because we have relatively substantial knowledge of naturally occurring patterns of *in vivo* synaptic input. To test our hypothesis, we must have knowledge of natural patterns of the hyperpolarization that “primes” the T-type calcium channels as well as the retinal excitation that activates them. T-type channels in LGN become deinactivated (“primed”) by visually driven hyperpolarization that can last several hundred ms, and they can then be activated by rEPSPs (Figure 1A) (Wang et al., 2007). The hyperpolarization is caused by GABA-mediated synaptic inhibition from local interneurons. Whereas rEPSGs provide evidence for a neuron's “preferred stimulus,” this “opponent inhibition” provides evidence against it. Thus, an ON-type TC neuron is excited by light in the center of its receptive field via rEPSGs, and inhibited by dark in the same region of retinotopic space via feedforward IPSGs from an OFF-type interneuron (Wang et al., 2011).

Excitatory drive is relatively simple because most TC neurons receive strong synaptic excitation from just one retinal neuron, which causes virtually all of its spikes (Usrey et al., 1998; Sincich et al., 2007; Weyand, 2007). Although rEPSGs show powerful paired-pulse depression (PPD) under standard slice conditions (following a long-period without presynaptic spikes, and in 2.0 mM extracellular calcium) (Chen et al., 2002; Blitz and Regehr, 2003), unitary rEPSG amplitude varies little over time in LGN *in vivo* (Carandini et al., 2007; Sincich et al., 2007; Borst, 2010), as suggested by inspection of Figure 1A (Wang et al., 2007). Although unitary rEPSG amplitude varies little, temporal summation has a profound influence on rEPSP amplitude and spike generation (Usrey et al., 1998; Carandini et al., 2007; Sincich et al., 2007; Weyand, 2007; Casti et al., 2008). Following a long interval without a retinal input spike (>50 ms), a single rEPSG rarely caused an output spike (1–10%), but when two input spikes occurred within 10 ms of one another, the probability of an output spike rose to about 90% (Sincich et al., 2007; Weyand, 2007). Although a large variety of other factors are undoubtedly important to the I-O relation, temporal summation of rEPSPs alone is sufficient to account for its general characteristics (Carandini et al., 2007; Casti et al., 2008). Thus, we consider “the input” to be the number of unitary rEPSGs, of approximately equal amplitude, that occur within a brief enough time to sum together in the postsynaptic potential.

### The homeostatic I-O relation

The optimal and homeostatic I-O relation can be derived from theory given sufficient knowledge of natural patterns of input (Fiorillo et al., 2014). Alternatively, the actual I-O relation can be characterized experimentally in response to natural patterns of stimuli, and it can then be inferred that this I-O relation is probably close to optimal on the assumption that neural systems in healthy adult animals should be well adapted to their environments. Both theory and experiment indicate that the homeostatic relation in TC neurons of LGN is one output spike for every 2 input spikes. The experimental evidence comes from simultaneous extracellular recording of rEPSPs and spikes from a single electrode and a single thalamocortical neuron (Kaplan et al., 1987; Movshon et al., 2005; Carandini et al., 2007; Sincich et al., 2007; Weyand, 2007; Casti et al., 2008). We have previously summarized and discussed the results of these studies in relation to the theory of predictive homeostasis (Fiorillo et al., 2014).

Visually driven opponent inhibition will hyperpolarize the membrane (Figure 1A) and will naturally cause a large reduction in membrane excitability in which rEPSGs will virtually never cause spikes (Figure 1B). According to theory, it is appropriate that sudden and unexpected evidence against the neuron's stimulus (an opponent-type IPSG) should strongly suppress the ability of a rEPSG to cause a spike and thereby drive the I-O relation away from its homeostatic level. However, if strong synaptic inhibition persists in a partially predictable manner, then it would be desirable for homeostatic mechanisms to restore the ability of rEPSGs to cause spikes.

During sustained hyperpolarization (−80 mV), T-type channels will deinactivate over hundreds of milliseconds and will then amplify rEPSPs. Relative to the homeostatic I-O relation associated with the depolarized state (−65 mV), the amplification must either be too weak, too strong, or “just right” (Figures 1B,C) Without amplification, rEPSGs will fail to cause spikes (assuming that rEPSGs at −80 mV and −65 are of similar amplitude), but if amplification is too strong, one input spike will consistently cause one or more output spikes, as implied by the “burst hypothesis.” If T-type channels are finely tuned, in accord with theory, the I-O relation will recover to near its homeostatic level (Figures 1B,C).

### Graded depolarization by T-type channels

We sought to mimic natural *in vivo* conditions using dynamic clamp patch recording in TC neurons in slices of rat dorsal LGN (Materials and Methods). Before considering EPSGs, we first examined responses to brief current injections with a square-wave pattern (10 ms, 100–900 pA in 10–50 pA increments), since this simpler waveform allowed us to more easily recognize and characterize the LTS. We studied voltage responses to currents injected from near −65 and −80 mV. To mimic the synaptically mediated hyperpolarization that occurs naturally during vision (Figure 1A) we used dynamic clamp to inject an artificial hyperpolarizing conductance for 1.2 s during each 3.2 s “sweep” (Figure 2A). The artificial conductance was 10 nS in each cell, but its reversal potential was adjusted as needed in each cell (range −85 to −100 mV) to hyperpolarize the membrane to near −80 mV. This conductance should ideally mimic the summed conductance associated with both the IPSG that causes the hyperpolarization and the rEPSG that activates T-type channels, as well as other synaptic conductances. We provide a justification for the artificial conductance amplitude in Materials and Methods.

We examined the extent to which T-type depolarizations (TTDs) were graded. A TTD was classified as a LTS if there was a region of positive slope in membrane voltage following offset of the depolarizing current (Figures 2B,C). As expected, the LTS emerged only after a few hundred ms at −80 mV, and it was blocked by nickel (400 μM) (Figure 2A). Among 22 total neurons, a LTS was observed in 22, 21, and 18 after 800, 400, and 200 ms, respectively, but not in any neurons at 50 ms.

There were two respects in which the TTD was graded rather than all-or-none. First, the “minimal LTS” (evoked by the minimum suprathreshold current) often caused 0 or 1 sodium spike, and larger currents evoked more spikes (Figures 2A–C). After 800 ms at −80 mV, the minimal LTS caused 0, 1, 2, and 3 spikes in 3, 8, 10 and 1 neuron, respectively. Under natural conditions, it is likely that most TTDs occur after a shorter duration of hyperpolarization to a less extreme potential. The minimal LTS caused 0 and 1 spike in 4 and 2 neurons after 100 ms at −80 mV (3 others had no LTS). After hyperpolarization to −75 mV for 800 ms, the minimal LTS caused 0 and 1 spike in 6 and 3 neurons, respectively (2 others had no LTS). This suggests that a large fraction of LTSs may cause no spikes, and thus greater synaptic excitation is likely to increase the number of spikes.

Second, TTDs were graded in amplitude for currents that were insufficient to cause a LTS. Even at 50 ms, the depolarization decayed faster in the presence of nickel (400 μM), indicative of a small TTD (Figure 2D). To isolate TTDs, we subtracted voltage responses at 50 ms from those at 100–800 ms (Figure 2C). At 100 ms, the largest current injection that did not cause a sodium spike generated a TTD of 9.0 mV (range 2.8–15 mV; *n* = 9) (Figure 2E). To characterize subthreshold TTDs, we identified the smallest current necessary to evoke a sodium spike in each cell, and we then averaged voltage responses across all cells at this threshold current and all smaller currents. At 800 ms, the threshold current evoked a TTD (which was a LTS) much larger than with just 10 pA less (Figures 2F,G). Nonetheless, subthreshold current injections caused TTDs that were graded in amplitude up to about 10 mV (Figures 2F,G).

TTDs were graded over a larger range of currents and voltages after shorter periods of hyperpolarization, which was due at least in part to a higher LTS threshold voltage when T-type channels were only partially deinactivated (Figures 2F,H). We were able to measure LTS threshold voltage with high precision due to our brief currents and small increments (the difference in peak depolarization to currents of 10 vs. 20 pA below the minimal current that evoked a LTS at 800 ms was 0.50 ± 0.34 mV, mean ± SD). The LTS threshold was ~5 mV more depolarized at 100 than 800 ms (−63 vs. −68 mV) (Figure 2H), and some neurons had no LTS at all at early times (see above). Likewise, LTS threshold was ~5 mV more depolarized when evoked from −75 mV (Figure 2H). In contrast, when the LTS was evoked in a more conventional experimental design, after at least a few seconds of sustained hyperpolarization and in the absence of any artificial conductance (Llinás and Jahnsen, 1982; Jahnsen and Llinás, 1984; Zhan et al., 1999; Gutierrez et al., 2001), LTS threshold was substantially more hyperpolarized (−72.5 ± 0.5 and −74.8 ± 0.5 mV, mean ± s.e.m., *n* = 30, when evoked from −80 and −90 mV, respectively).

A notable feature of the LTS is its variable timing. When it is evoked by minimal supra-threshold currents, or when only a small fraction of T-type channels are deinactivated and able to contribute to the LTS, the peak of the LTS occurs substantially later, by more than 100 ms in some cells (Figures 2B,C) (Zhan et al., 1999). This would appear problematic if it were to create a corresponding level of variability in spike timing (relative to rEPSGs). However, subthreshold TTDs (those with no LTS) reached peak amplitude at 28 ± 4.5 ms after current onset (mean ± SD at 200 ms), and the time course varied little with current amplitude or time at −80 mV (Figure 2F) or across cells (Figure 2E). We did not observe spikes at latencies longer than the peak of subthreshold TTDs (>30 ms) (see Spike Timing).

To examine the effect of the TTD on excitability we measured the minimal (“threshold”) current required to evoke at least one sodium spike. Relative to the baseline threshold current at −65 mV, the current was twice as great at 50 ms, but it recovered by 200 ms and at 800 ms it was 81 ± 22% (mean ± SD) of baseline (Figure 3) (*n* = 17, *p* = 0.005, paired *t*-test). Excitability did not recover when T-type channels were blocked with nickel (400 μM) (Figure 3). Despite the modest increase in excitability observed after 800 ms, excitability was much closer to its homeostatic level (that observed at −65 mV) because of the presence of T-type channels (Figure 3).

### T-type depolarization restores sensitivity of spike output to synaptic excitation

Above we have provided evidence that the TTD functions in a graded manner, but only over a moderately narrow range of input amplitudes. The theory of predictive homeostasis implies that this narrow range will be closely matched to the range of naturally occurring rEPSP amplitudes. We therefore studied responses to rEPSGs, as well as artificial EPSGs (aEPSGs) delivered via dynamic clamp (Materials and Methods). We were guided by knowledge of naturally occurring patterns of synaptic input (see “The Homeostatic I-O Relation”), particularly the finding that unitary rEPSG amplitude displays only modest variation, and temporal summation of 2 rEPSGs is usually required to cause 1 spike (Sincich et al., 2007; Weyand, 2007).

Synaptic events consisted of 1, 2, or 4 EPSGs delivered with a standard inter-EPSG interval of 5 ms. rEPSGs were studied in the presence of a low concentration of GABA_B_ agonist (0.05–1.0 μM baclofen) to help remove the unnaturally high level of paired-pulse depression found under standard slice conditions, and to thereby better reproduce the temporal summation of rEPSPs that is necessary to cause spikes *in vivo* (see Materials and Methods). At the start of recording from each cell tested with aEPSGs, the amplitude of unitary aEPSGs was adjusted so that temporal summation of 2 aEPSGs (separated by 5 ms) was necessary to cause 1 spike from a potential of −65 mV. This method resulted in aEPSG and aEPSP amplitudes almost identical to their retinogeniculate counterparts (see Materials and Methods). The similarity was remarkable given that we selected aEPSG amplitudes to reproduce *in vivo* I-O relations, rather than aiming to directly match rEPSG amplitude. The similarity helps to validate our method of attempting to mimic natural conditions.

EPSG events were delivered at −65 mV and at various times after application of an artificial conductance that hyperpolarized the membrane to near −80 mV (Figures 4A,B). As expected, EPSP amplitude increased over time at −80 mV (Figures 4A–C). We isolated TTDs in the same way described above, by subtraction of the voltage waveform after 50 ms at −80 mV from that at 100–800 ms. At 800 ms, the TTD was roughly comparable in average peak amplitude to the EPSP at 50 ms (~10 mV), although it had a slower time course (Figure 4C). Similar to results with square-wave current pulses (Figure 2), the time course of TTDs varied little with time at −80 mV or with number (or frequency; not shown) of temporally summed EPSGs (Figure 4C).

As at −65 mV, temporal summation of EPSGs was necessary for spike generation from −80 mV. For illustration only, we sorted the 26 neurons into three color-coded groups based on their responses to one aEPSG at 800 ms (Figure 4D, lower left). One aEPSG caused virtually no TTD in half the cells (13 of 26), but a large TTD in the other half (Figure 4D) (this could potentially reflect functional diversity of cell types, perhaps with differing receptive fields, but it is more easily explained by the natural variation found within nominally homogeneous cell populations, as well as artifactual variation due to slice preparation, together with some all-or-none character of the TTD). In cells with no TTD in response to one aEPSG, temporal summation with a second aEPSG caused a large TTD in all of these cells (13 of 13). However, large TTDs often failed to cause spikes. In response to one aEPSG, 6 of 13 cells with large TTDs had no spike, but all 6 of these cells had at least one spike in response to two aEPSGs (Figure 4D, lower right). Similar results were observed after 200 ms (Figure 4D, upper traces) and for rEPSGs. Thus, generation of TTDs often depended on temporal summation of two EPSPs, and even in the presence of a TTD, additional EPSGs were often required to cause spikes. Consistent with theory, both TTD generation and spike generation were highly sensitive to EPSG input count.

**Figure 5:**
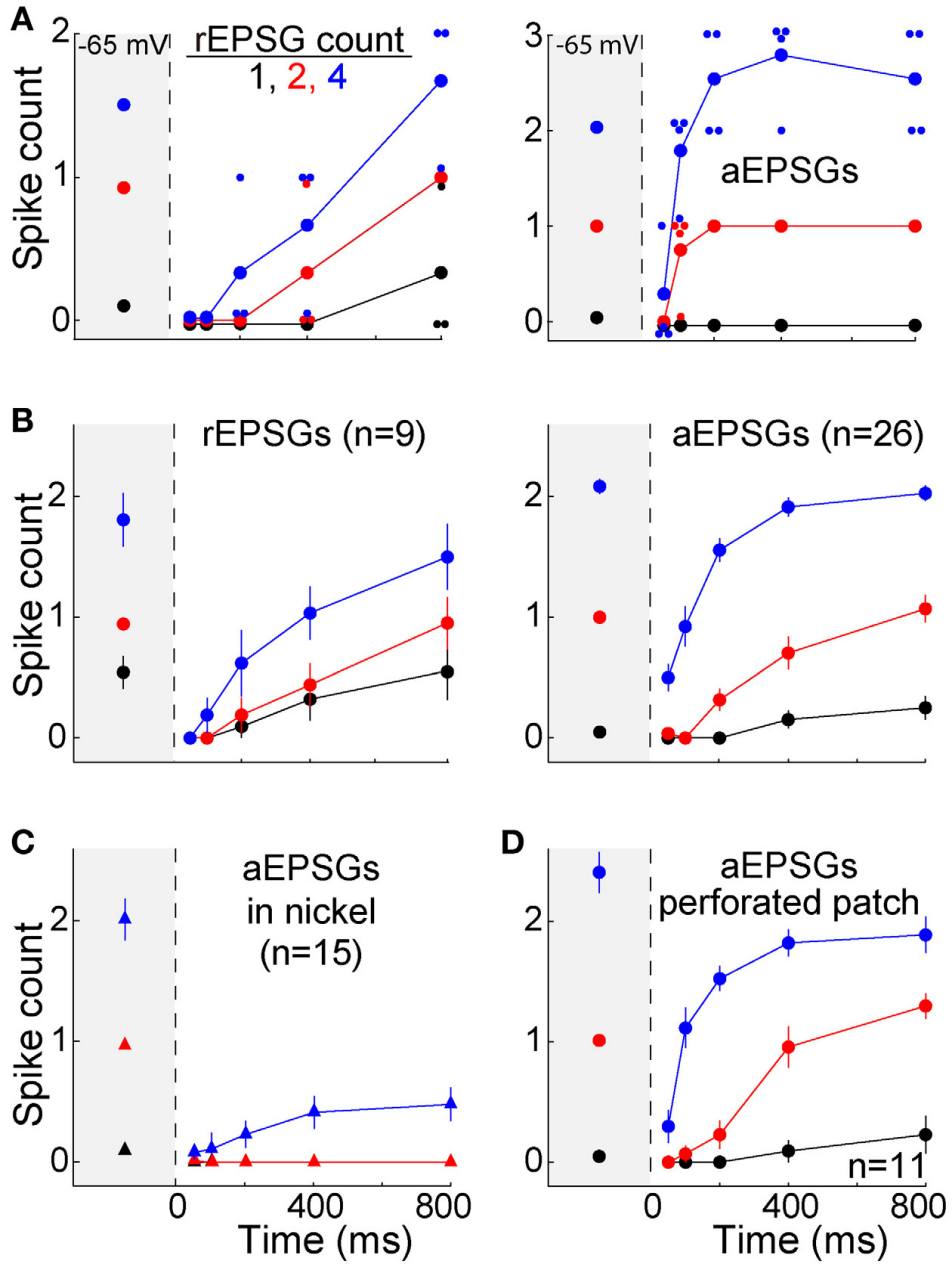
**Recovery of spike counts following hyperpolarization**. Spike counts (mean ± s.e.m.) in response to 1 (black), 2 (red), and 4 EPSGs (blue) at −65 mV and as a function of elapsed time at −80 mV. **(A)** Spike counts in response to rEPSGs (left) and aEPSGs (right) in two individual neurons. Large symbols represent means, and small symbols represents spike counts for a single EPSG event, offset slightly in some cases to avoid overlap with other data points. The cell illustrating aEPSGs was chosen because it had the highest spike counts at 800 ms among 26 neurons. **(B)** Spike counts (mean ± s.e.m.) in response to rEPSG (left) and aEPSG (right) events averaged across the population of recorded neurons. Of 26 neurons tested with aEPSGs, only 13 were tested at 100 ms. **(C)** Spike counts in response to aEPSGs did not recover in the presence of nickel (300 μM). **(D)** Population spike counts in response to aEPSG events, as in recordings in the whole cell configuration in **(B)**, but for a separate population of 11 neurons recorded with perforated-patch pipettes.

The TTD was found to increase excitability toward but not above its homeostatic level (Figures 5A,B). The single cell shown with aEPSGs (Figure 5A, right) displayed moderately greater excitability at −80 than −65 mV (in the case of 4 aEPSGs after 200 ms or more). However, we chose to illustrate this cell because it had the greatest number of spikes at 800 ms of any of the 26 cells tested. Nonetheless, it never exhibited a spike count that exceeded the number of aEPSG inputs, and in this sense it never exhibited internally driven bursts of spikes. Thus, consistent with theory, we never observed a cell with excitability at −80 mV that was substantially above the homeostatic ideal. Averaged across the population of all neurons, the number of spikes at −80 mV recovered to but not above the level observed at −65 mV by 800 ms, either with real or artificial EPSGs (Figure 5B).

As expected, recovery did not occur in the presence of nickel (300 μM) (Figure 5C). This concentration of nickel would not be expected to fully block T-type calcium channels, and it is likely to have partially blocked other types of calcium channels (200 μM nickel blocked 66% of T-type current and 36% of L-type current in thalamocortical neurons of ventrobasal thalamus; Huguenard and Prince, 1992). However, the recovery of excitability observed here matches the well-known voltage-dependence and kinetics of T-type channels. Furthermore, our hypothesis is not exclusive to T-type channels, and thus other channels may also contribute to the recovery of excitability during hyperpolarization.

The solution within our whole-cell patch pipettes could have altered the function of T-type channels or other aspects of membrane excitability. However, similar results were observed with perforated-patch recordings (*n* = 12) (Figure 5D). In separate experiments with whole-cell recordings that were designed to test stability (*n* = 5), no significant change was observed in the TTD or membrane excitability over more than 15 min (which was longer than the period of ~10 min required for our standard protocol). This is consistent with previous evidence that T-type currents are stable in whole-cell recordings (Kuo and Yang, 2001). Thus, the lack of an all-or-none burst of spikes was unlikely to have been an artifact of whole-cell recording.

**Figure 6:**
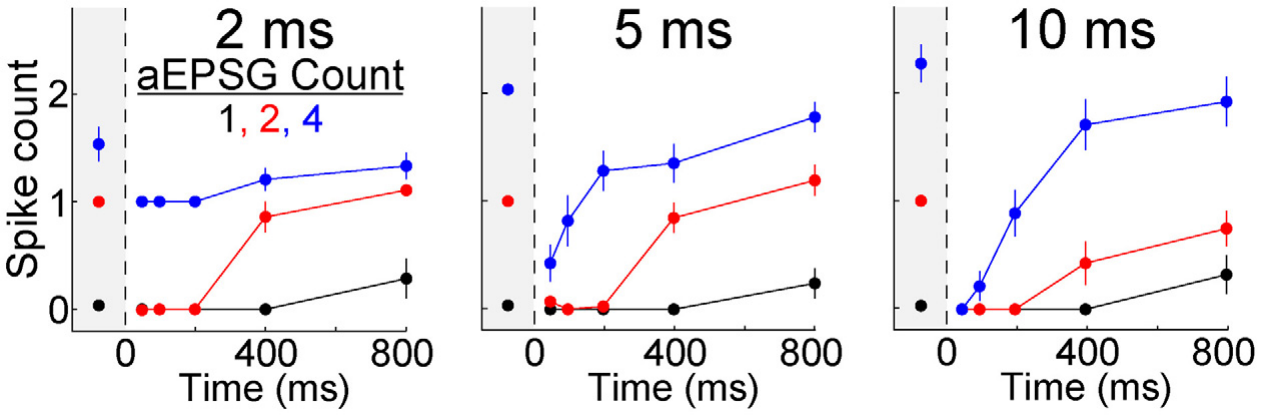
**Recovery of spike counts in response to events with inter-aEPSG intervals of 2, 5, and 10 ms**. Fully analogous to Figure 5B with 5 ms intervals, but for a separate population of 7 neurons that were also tested with 2 and 10 ms intervals. Gray region at the left of each plot shows spike counts evoked from −65 mV. Prior to the protocol being run with a particular interval, aEPSG amplitude was adjusted as necessary so that the second of two aEPSGs at −65 mV would evoke one spike. Intervals were tested in the order 2, 5, and then 10 ms.

We asked whether the TTD would also have a homeostatic function for other aEPSG intervals (input frequencies). In 7 additional neurons tested with aEPSG intervals of 2, 5, and 10 ms, we obtained results qualitatively similar to those reported above for 5 ms intervals (Figure 6). The lack of excessive excitability in response to EPSG events with 2 ms intervals (500 Hz) is noteworthy, because the rapid and large depolarization would be expected to have maximal effect in eliciting an LTS and driving bursts of spikes. Our aEPSGs were designed with no “paired-pulse depression” (each was of identical conductance amplitude), and therefore the resulting aEPSPs may be as large and fast as any that would occur naturally.

**Figure 7:**
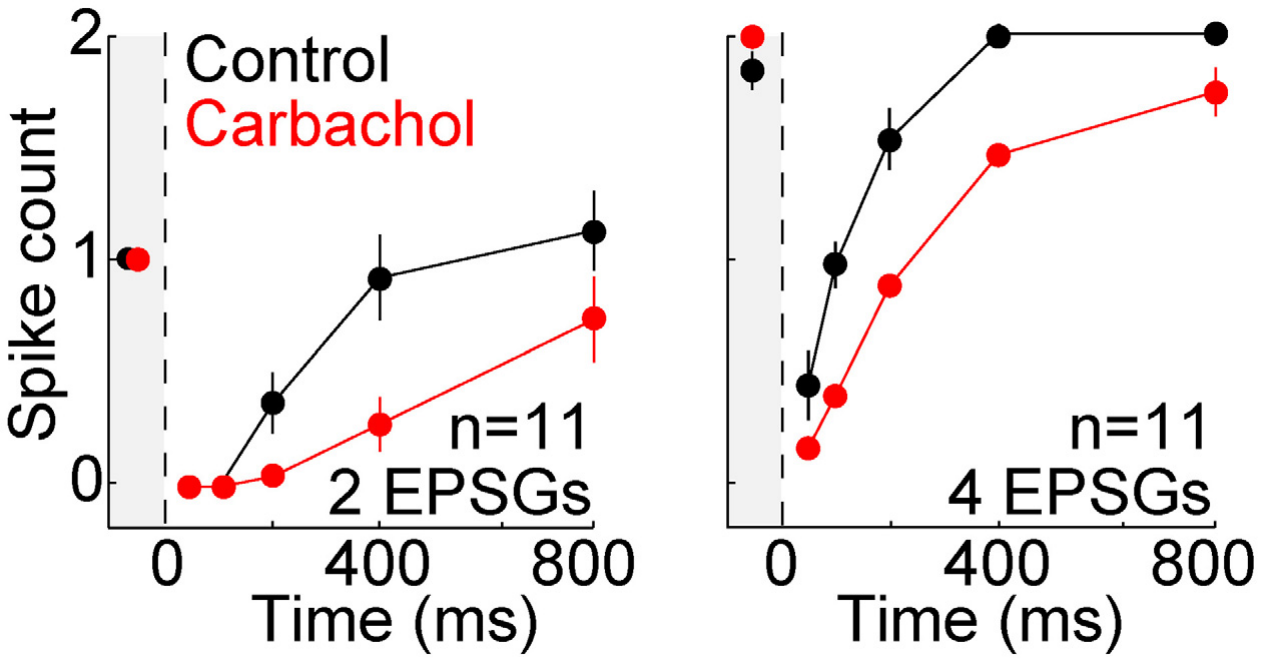
**Suppressed recovery of spike counts during activation of cholinergic receptors**. Mean (±s.e.m.) spike counts evoked by 2 (left) and 4 (right) aEPSGs in 11 neurons before (black) and after (red) perfusion of the cholinergic agonist carbachol (3–10 μM). Plots are analogous to Figure 5B, but with 2 and 4 aEPSGs shown on separate plots. From −65 mV, carbachol caused depolarization of 7.4 ± 3.4 mV and reduced membrane resistance by about 20 MΩ. Adjustments were then made once again, following the same method used at the start of each recording, so that 2 aEPSGs from −65 mV caused 1 spike.

Modulatory neurotransmitters are typically at higher extracellular concentrations in an awake brain compared to a brain slice, and acetylcholine is known to have a strong influence on TC neurons. We therefore studied 11 additional neurons in the presence of carbachol (3–10 μM), a cholinergic receptor agonist. The same protocol described above with aEPSGs was performed before and after perfusion of carbachol. As expected, carbachol caused depolarization (7.4 ± 3.4 mV). Membrane potential and aEPSG amplitude was then adjusted once again, in the same way that it was at the start of recording in each cell, so that 2 aEPSGs delivered from −65 mV caused one spike. Relative to control conditions, carbachol suppressed the recovery of spike counts over time at −80 mV (Figure 7). Subsequent perfusion of a muscarinic acetylcholine receptor antagonist (1 μM scopolamine) in the continued presence of carbachol partially reversed the suppressive effect of carbachol, although its effect was of borderline significance (*n* = 5, *p* = 0.11 and 0.02 for 4 aEPSGs at 400 and 800 ms, respectively, paired *t*-tests). These results might be explained by the finding that muscarinic receptor activation suppresses LTS amplitude (Zhan et al., 2000). Thus, cholinergic stimulation may blunt the effect of T-type channels, strengthening our conclusion that T-type channels work toward restoring excitability rather than increasing excitability above its homeostatic baseline.

### Maintenance of input-output relations

**Figure 8:**
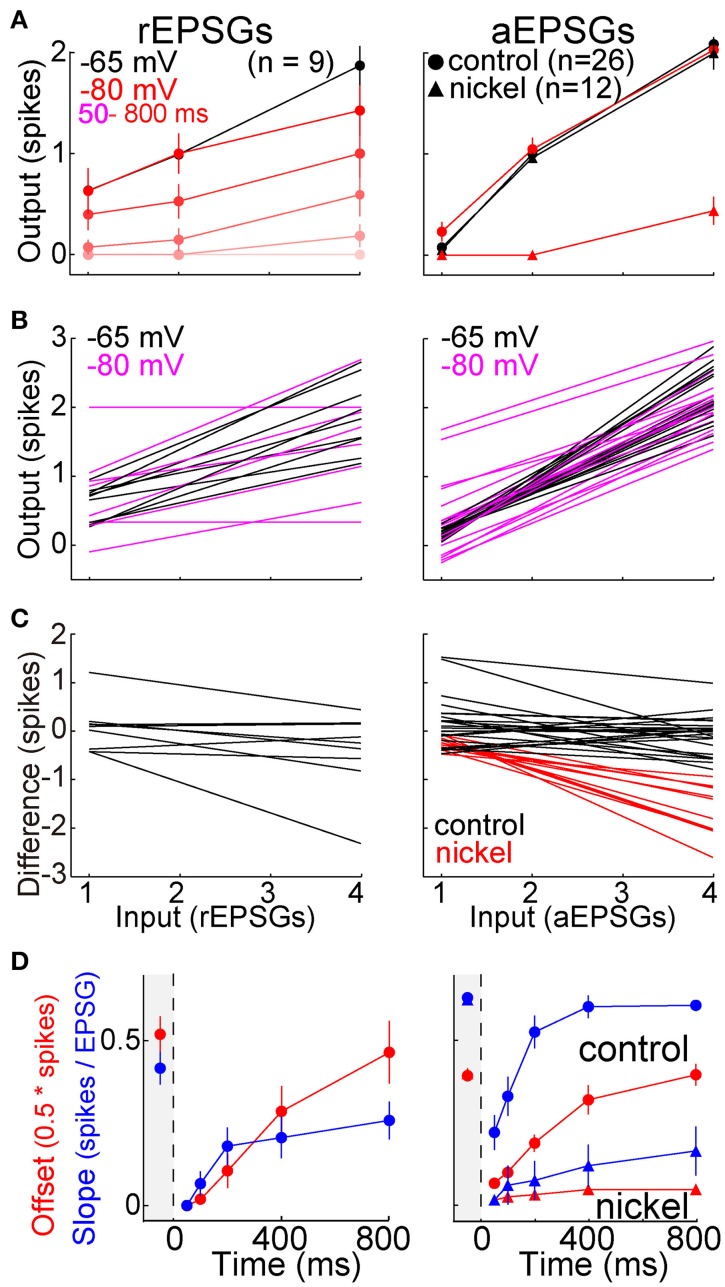
**Recovery of the I-O relation following hyperpolarization**. rEPSGs at left, aEPSGs at right. **(A)** Population average spike counts (mean ± s.e.m.) as a function of EPSG count at −65 mV (black) and at −80 mV (pink and red). For rEPSGs (left), spike counts are shown in response to rEPSGs at 50–800 ms following onset of hyperpolarization. For aEPSGs (right), spike counts are shown both in the presence (triangles) and absence (circles) of nickel (300 μM), and data at −80 mV is shown only for 800 ms. **(B)** A line was fit to mean spike counts (3 or 4 counts per cell) in each individual neuron at −65 (black) and after 800 ms at −80 mV (magenta). **(C)** The linear fits of **(B)** at −65 mV were subtracted from those at −80 mV to show how the I-O relation changed in each neuron. The preponderance of flat lines indicate little change in most neurons, whereas the negative slopes observed in the presence of nickel (red) indicate that the I-O relation at −80 mV had lower slope than at −65 mV. **(D)** Using the linear fits of **(B)**, the time course of recovery was characterized for both the slope and y-offset (mean ± s.e.m.). The y-offset was defined as the number of spikes in response to 2 EPSGs, and was multiplied by 0.5 only so that the scale was similar to that of slope for the purpose of illustration. Recovery was suppressed in the presence of nickel (triangles).

To further examine I-O relations, we counted spikes as a function of the number of EPSGs in an event. Averaged across the population of neurons, the I-O relation was close to linear with a slope near 0.5 at −65 mV (Figure 8A). Following hyperpolarization to −80 mV, the slope approached zero, but it recovered over time so that by 800 ms it was virtually indistinguishable from that at −65 mV (Figure 8A). Recovery was blocked by nickel (300 μM) (Figure 8A).

Figure 8B shows linear fits of I-O counts in each neuron at −65 and after 800 ms at −80 mV. To compare the two in each neuron, we subtracted the linear fit at −65 from the one at −80 mV. The difference had a slope near zero in most neurons (indicating recovery of homeostatic excitability) and was slightly negative in others (incomplete recovery) (Figure 8C). As expected, the differences were negative in the presence of nickel (Figure 8C). Although the I-O relation was restored over time at −80 mV, slopes tended to be slightly less than at −65 mV even after 800 ms, indicating that excitability did not fully recover in a substantial fraction of neurons (Figures 8B–D) (rEPSGs: 0.26 ± 0.06 at 800 ms vs. 0.42 ± 0.05 at −65 mV, *p* = 0.06, *n* = 9, paired *t*-test; aEPSGs: 0.59 ± 0.02 at 800 ms vs. 0.66 ± 0.2, *p* = 0.05, *n* = 26).

**Figure 9:**
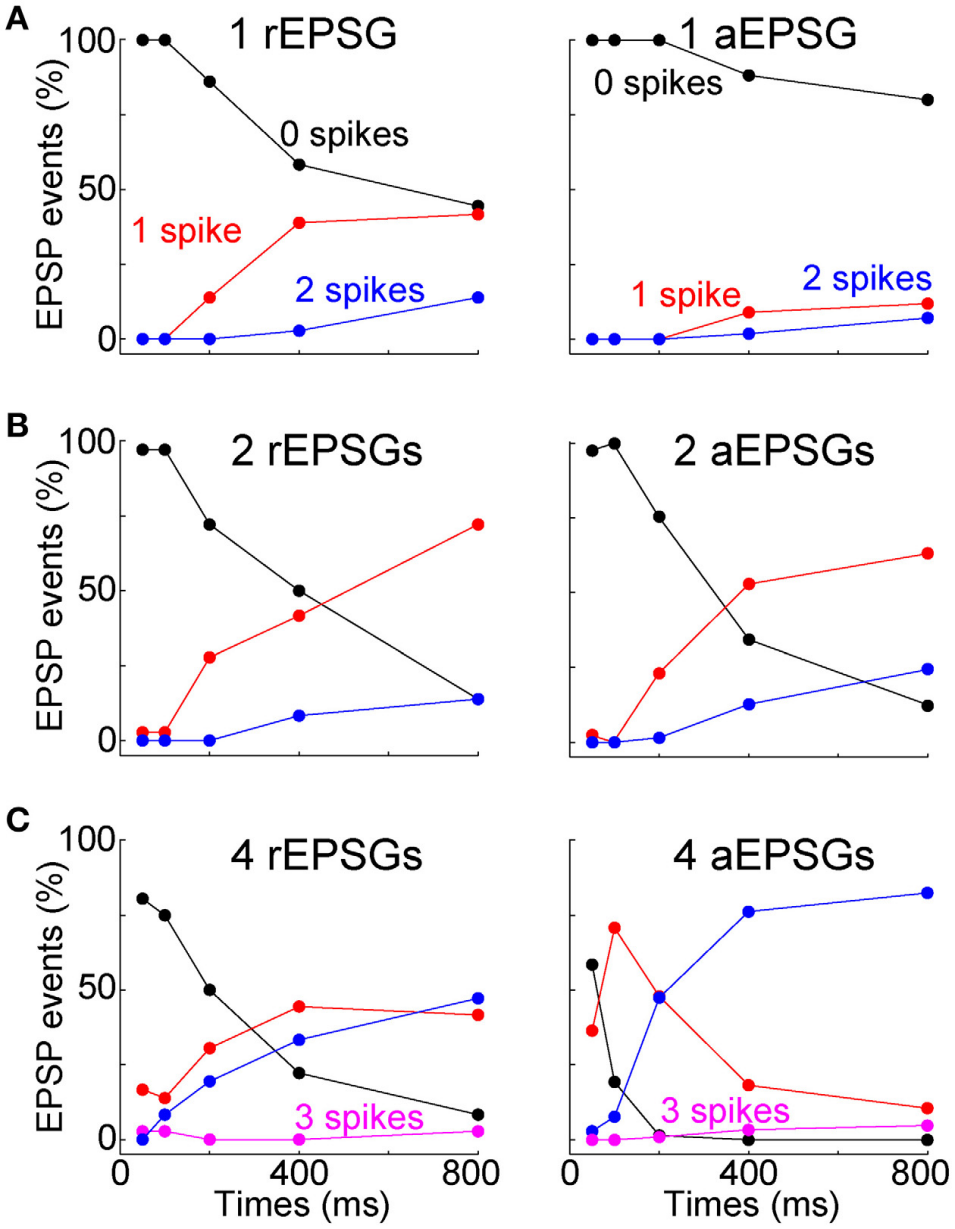
**Rarity of excess spikes**. The fraction of all EPSG events (rEPSGs at left, aEPSGs at right) that resulted in 0 (black), 1 (red), 2 (blue), and 3 (magenta) spikes as a function of time at −80 mV for **(A)** 1 EPSG, **(B)** 2 EPSGs, and **(C)** 4 EPSGs. Spike count only exceeded EPSG count in a small fraction of cases in which 1 EPSG caused 2 spikes at 400 and 800 ms. For each EPSG count and time point, there were a total of 27 rEPSG events (3 per cell in 9 cells) and 260 aEPSG events (4 per cell in 65 cells), except 208 aEPSG events at 100 ms (4 per cell in 52 cells). For aEPSG events, data includes all cells recorded under standard conditions (2.0 mM calcium) with 5.0 ms inter-aEPSG intervals, including perforated-patch recordings.

Most above analyses have examined mean spike counts. For the burst hypothesis to be correct would not necessarily require that the mean spike count is increased above the level observed at −65 mV. It is conceivable that the mean could be preserved but the distribution changed so that most EPSG events result either in zero spikes or in a burst of multiple spikes. We therefore examined all I-O counts from all individual EPSG events in all cells (under control conditions) (Figure 9). Out of a total of 2184 aEPSG events in 65 neurons at 50–200 ms (1, 2, and 4 aEPSGs), we never observed an excess spike (O/I never exceed 1). Nor did we ever observe an excess spike in response to 2 or 4 aEPSGs at 400 or 800 ms (among 1040 events). The only excess spikes occurred with 1 aEPSG at 400 and 800 ms, where we found 21 of 520 events with 2 spikes (4%, or 0.6% of 3744 total events across all 3 aEPSG counts at all 5 time points). We did not observe a single instance of any EPSG event causing an excess of 2 or more spikes (0 of 3744 events). Virtually identical results were found among a total of 405 rEPSG events in 9 cells (Figure 9). Thus, we conclude that T-type driven bursts were exceedingly rare.

### T-type channels cause excessive excitability in a low conductance state

**Figure 10:**
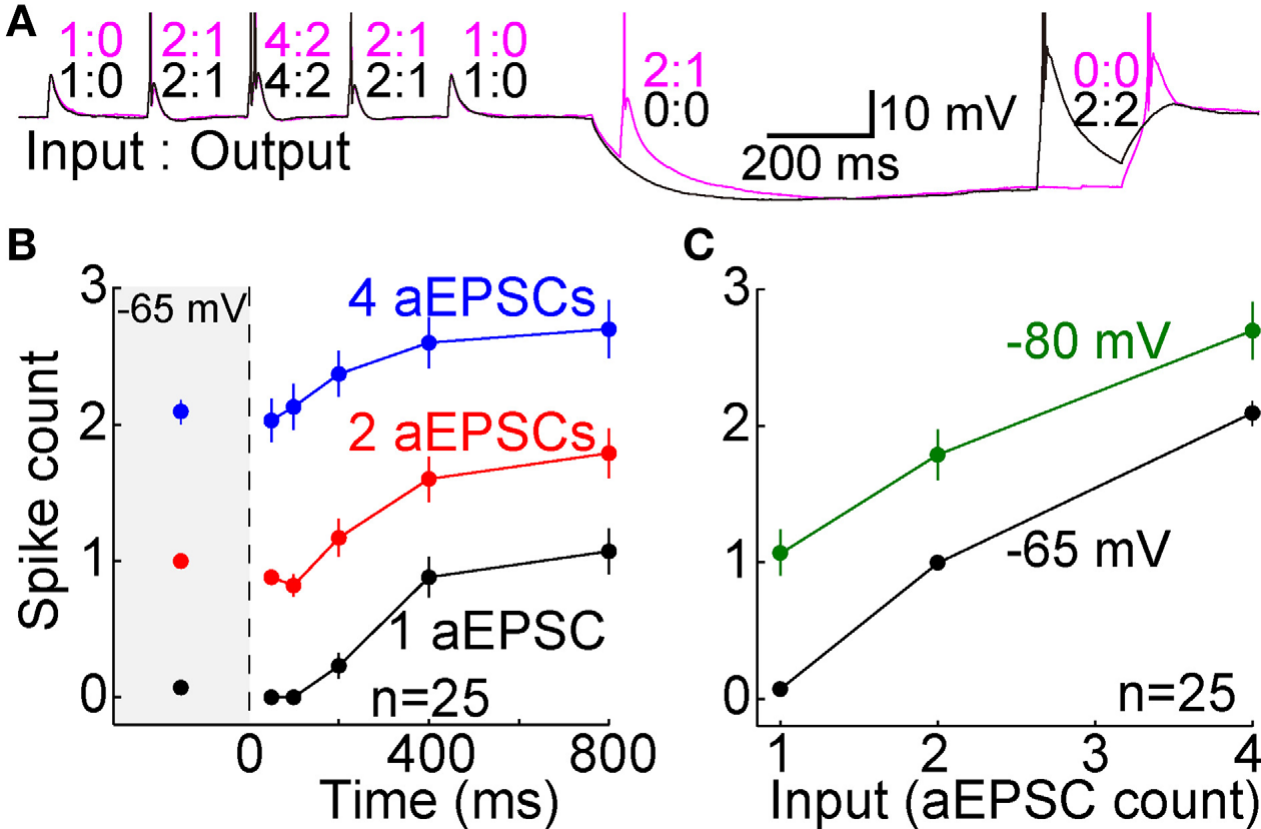
**T-type channels cause excessive spike counts when activated in a low conductance state**. **(A)** Voltage recording from a single cell. Fully analogous to our standard aEPSC experiment shown in Figure 4B, but performed without artificial conductances. Both hyperpolarization and aEPSPs were achieved solely by current injection (aEPSCs in “current clamp” rather than aEPSGs in “dynamic clamp”). The higher membrane resistance may account for the slower hyperpolarization (relative to Figures 4A,B), which was not complete after 50 ms. Two sweeps are shown, one with two aEPSCs at 50 ms (magenta) and another with two aEPSCs at 800 ms (black). Inset numbers indicate aEPSC input and spike output for each event. **(B)** Compare to Figure 5B. After 200–800 ms at −80 mV, spike count (mean ± s.e.m.) exceeded its homeostatic level at −65 mV in response to 1 (black), 2 (red), and 4 aEPSCs (blue), in a separate population of 25 neurons that contributed data only to this figure. **(C)** Average spike counts (mean ± s.e.m.) as a function of EPSG count at −65 mV (black) and after 800 ms at −80 mV (green). Compare to Figure 8A.

The same experiment (as in Figure 4B) was repeated in a separate group of neurons (*n* = 25), but the hyperpolarization to −80 mV and the aEPSPs were caused only by current injection, without any mimicry of synaptic conductances (Figure 10A). Following hyperpolarization, excitability recovered to a significantly higher level than either at −65 mV (Figure 10B), or after 400 or 800 ms in experiments with artificial conductances (*p* < 10^−6^ in all cases, paired and unpaired *t*-tests). Likewise, the I-O relation after 800 ms at −80 mV was shifted upwards relative to that at −65 mV (Figure 10C). These results suggest that the suppressive effect of synaptic conductances on membrane excitability (via shunting) is an important factor in the I-O relation, which potentially explains why studies that used only current injection found that T-type channels caused a stereotyped burst of spikes (Llinás and Jahnsen, 1982; Jahnsen and Llinás, 1984; Zhan et al., 1999; Gutierrez et al., 2001).

### Spike timing

**Figure 11:**
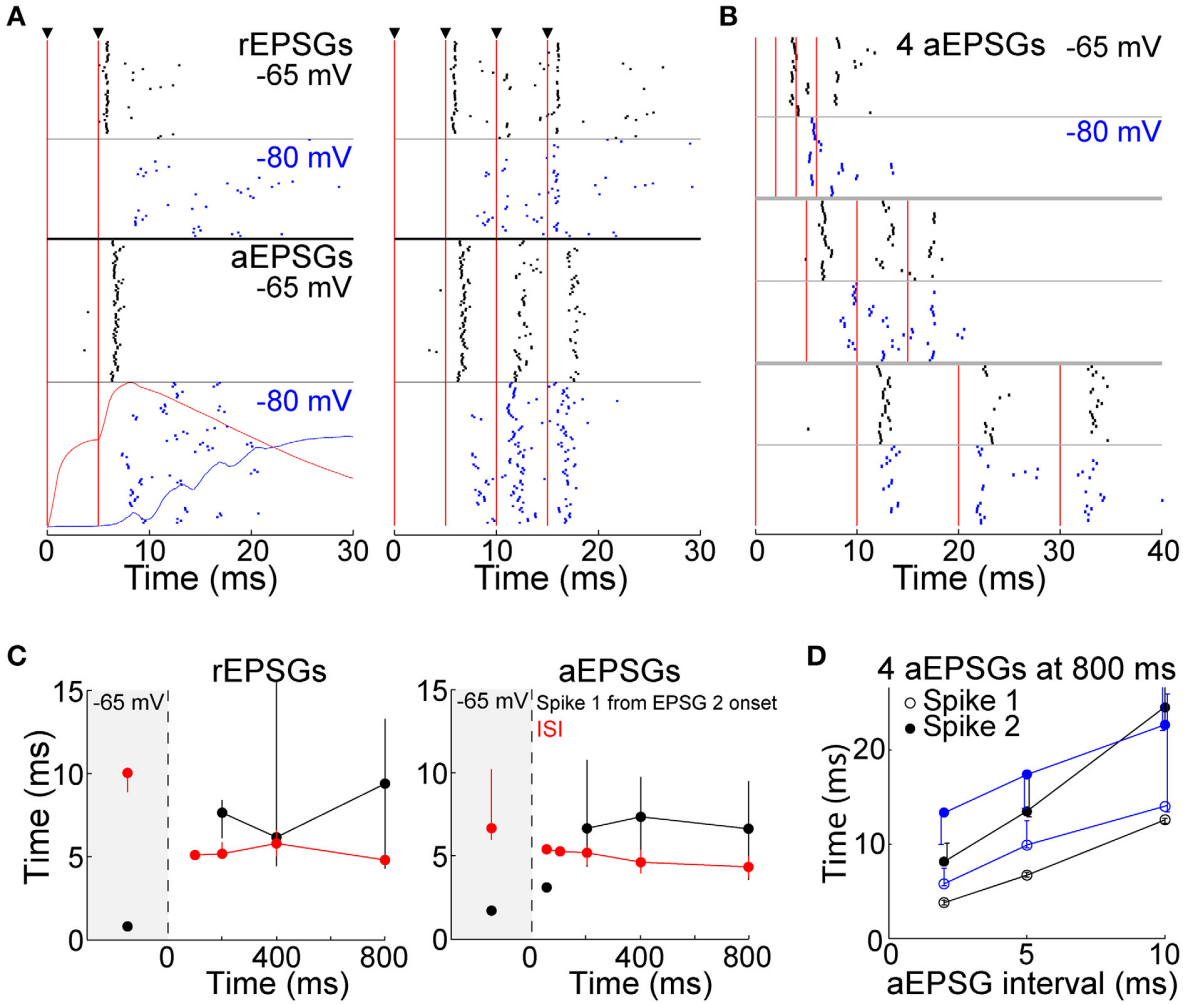
**Spike timing. (A)** Rasters of spike times in response to 2 (left) and 4 EPSGs (right) delivered from −65 (black) and −80 mV (blue), with rEPSGs at top (6 trials from each of 9 cells, half at 400 ms and half at 800 ms) and aEPSGs at bottom (4 trials in each of 26 cells at 800 ms). Vertical red lines indicate EPSG onsets. For 2 aEPSGs at −80 mV, the population average aEPSP at 50 ms (red) and TTD at 800 ms (blue), (identical to those in Figure 4C) are shown for comparison. **(B)** Spike times from 7 cells tested with inter-aEPSG intervals of 2, 5, and 10 ms. **(C)** First spike times (in response to 2 EPSGs, black) and first ISIs (in response to 4 EPSGs, red) at −65 mV and as a function of time at −80 mV, with rEPSGs at left and aEPSGs at right. EPSGs were delivered with 5 ms intervals as in **(A)**. Medians (±25 percentiles) are from all spikes in all cells. The number of spikes range from 2 at 50 ms to 1559 at −65 mV for aEPSGs, and 3–387 for rEPSGs. **(D)** Times of first and second spikes evoked from −65 mV (black) and after 800 ms at −80 mV (blue), measured from onset of first aEPSG, as a function of aEPSG interval. Median times (±25 percentiles) of 5–28 spikes at −80 mV, and 260–420 spikes at −65 mV. For the case of the second spike with an inter-aEPSG interval of 10 ms, the 75th percentile was truncated for clarity; it was at 33 ms, whether evoked from −65 or −80 mV. Similar to what is shown here at 800 ms, spike times also increased with aEPSG interval at 200 and 400 ms (not shown).

Whereas above we show that T-type channels help to preserve spike counts, here we investigate their effect on spike timing. We presume that each spike should ideally be caused by a rEPSG, preferably with a delay as brief as possible. However, it is difficult to imagine an amplification mechanism that could preserve at −80 mV the spike timing seen at −65 mV while also preserving I-O counts. The median latency of spikes *in vivo* is 1–3 ms (Usrey et al., 1998; Sincich et al., 2007; Weyand, 2007). We observed similar latencies to the first spike from −65 mV, but latencies were substantially longer when EPSGs were delivered from −80 mV (7.7 ± 1.0 ms at −80 mV vs. 1.7 ± 0.1 ms at −65 mV, measured from onset of the second of two aEPSGs). The raster of Figure 11A shows spike times across all cells (4–6 trials per cell). The variability in timing of the first spike (“jitter”) appears greater at −80 mV than −65 mV (Figure 11A), but this was primarily due to variability across cells rather than across trials in individual cells. When measured by the coefficient of variation (SD/mean), variability in spike timing across trials in individual cells was modestly reduced at −80 mV (0.08 ± 0.01 at −80 mV vs. 0.21 ± 0.06 at −65 mV for cells tested with 2 aEPSGs or rEPSGs, *p* = 0.036, *n* = 29, paired *t*-test). Short inter-spike intervals (ISIs) are known to be a hallmark of T-type-induced bursts, and indeed, intervals were modestly reduced at −80 mV (Figure 11C).

The effect of hyperpolarization on spike timing was mostly restricted to the early period of high frequency EPSG events, as would be expected given temporal summation of EPSGs and a graded TTD. Spikes followed a third and fourth EPSG with shorter latencies (than a second EPSG) (Figures 11A,B). Spike latency was lower following the second of two aEPSGs when the aEPSG interval was 10 ms rather than 5 ms (Figures 11B,D). Likewise, ISIs were longer for 10 ms than 5 ms inter-aEPSG intervals (*p* < 10^−5^ for ISIs at 5 vs. 10 ms, rank sum test).

Although spike timing was altered at −80 mV, EPSGs rather than TTDs remained the primary and proximal cause of spikes. With 5 ms EPSG intervals, virtually all spikes occurred within 15 ms after onset of the second EPSG, substantially before the peak of the TTD at about 25 ms (Figures 4C, 11A). Furthermore, as the TTD increased in amplitude over time at −80 mV (Figure 4C), there was no change in either ISI or first spike latency (Figure 11C). Finally, in 7 neurons tested with aEPSG intervals of 2, 5, and 10 ms, spike times were clearly related to timing of aEPSGs (Figures 11B,D). This was particularly apparent for aEPSG intervals of 10 ms, in which case there was little difference between −80 and −65 mV (Figures 11B,D). Thus, TTDs were not associated with stereotyped spike timing, and the causal link of synaptic input and spike output was substantially preserved.

### Calcium concentration

**Figure 12:**
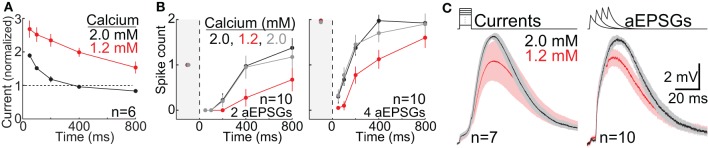
**Reduced recovery of excitability in 1.2 vs. 2.0 mM calcium**. Our standard protocols were performed in 2.0 mM followed by 1.2 mM calcium in 17 neurons. **(A)** In the square-wave protocol (Figure 2), recovery of excitability at −80 mV was reduced in 1.2 mM (red) vs. 2.0 mM calcium (black), as measured by the minimal “threshold” current required to evoke a sodium spike. Plot is analogous to Figure 3. Currents in each cell were divisively normalized to the corresponding threshold current at −65 mV (386 ± 25 pA in 2.0 mM calcium, 245 ± 39 pA in 1.2 mM calcium, mean ± s.e.m.). **(B)** In the aEPSG protocol (Figure 4), recovery of spike counts at −80 mV was reduced in 1.2 mM calcium (2 aEPSGs at left, 4 aEPSGs at right). The protocol in each cell was performed sequentially in 2.0 (black), 1.2 (red), and again in 2.0 mM calcium (gray). **(C)** Population TTDs (mean ± s.e.m.) in response to square-wave current (left) and aEPSG inputs (right). The input conditions (current or aEPSG count) in each cell were selected to maximize TTD amplitude in the absence of sodium spikes, and were averaged across times at −80 mV (100–800 ms) (see Materials and Methods). TTDs were evoked in 2.0 and 1.2mM calcium in each cell, and responses were normalized to the peak TTD amplitude in 2.0mM calcium prior to averaging across cells. One cell tested in the square-wave protocol was included here (and in the statistics presented in Results), but excluded in **(A)**, because of a large and sudden change in input resistance that occurred after collection of data at 50 and 800 ms in 1.2 mM calcium (thus data collected subsequently at 100, 200, and 400 ms in 1.2 mM calcium was discarded).

The results presented above were obtained with an extracellular calcium concentration of 2.0 mM, which is typical of *in vitro* experimental conditions, including those studies that found bursts with an all-or-none character (Llinás and Jahnsen, 1982; Jahnsen and Llinás, 1984; Zhan et al., 1999; Gutierrez et al., 2001). However, estimates of *in vivo* calcium are at least 25% lower (1.1–1.5 mM) (Hansen, 1985; Jones and Keep, 1988). To examine the effect of lower calcium, the same experimental protocols described above were performed in an additional group of neurons in 2.0 mM extracellular calcium, followed by perfusion of solution containing 1.2 mM calcium. Membrane potentials and aEPSG amplitudes were readjusted in 1.2 mM calcium in the same way that they were at the start of recording in each cell (see above). In 1.2 mM calcium, recovery of excitability over time at −80 mV was reduced, and was not complete by 800 ms, whether measured in the square-wave protocol (Figure 12A) or the aEPSG protocol (Figure 12B). The effect of 1.2 mM calcium was reversed by subsequent return to 2.0 mM calcium (Figure 12B). These results further strengthen our conclusion that T-type channels work toward restoring excitability rather than increasing excitability above its homeostatic baseline.

Lower external calcium would be expected to reduce TTD amplitude by reducing T-type calcium currents, and in principle this could explain the suppression of recovery at −80 mV. However, the reduction in external calcium had additional effects on excitability that made it difficult to determine the likely contribution of reduced T-type currents to the reduced recovery of excitability at −80 mV. Perfusion of 1.2 mM calcium resulted in a small but significant hyperpolarization (−1.6 ± 2.1 mV from −65 mV, mean ± SD, *n* = 17, *p* = 0.007, paired *t*-test), which was corrected by current injection. In addition, it decreased action potential threshold (−2.1 ± 1.3 mV, mean ± SD, *n* = 17, *p* = 0.0007) and increased membrane resistance (+11.5 ± 12.8% at −65 mV, mean ± SD, *n* = 13, *p* = 0.007). Likewise, the current required to cause a spike decreased, whether injected from −65 mV (386 ± 25 pA in 2.0 to 245 ± 39 pA in 1.2 mM, mean ± s.e.m., *n* = 7, *p* = 0.0001, paired *t*-test) or after 50 ms at −80 mV (730 ± 45 pA to 603 ± 92 pA, *p* = 0.0009). In contrast, there was an insignificant increase after perfusion of 1.2 mM calcium in the current required to cause a spike after 800 ms at −80 mV (344 ± 44 pA to 365 ± 68 pA, *p* = 0.14), suggesting that although excitability was generally greater in 1.2 mM calcium, this was compensated by a reduced T-type calcium current once T-type channels were deinactivated.

Despite the fact that an increase in membrane resistance would be expected to increase TTD amplitude, TTDs tended to be smaller in lower calcium (Figure 12C) (−3.4 ± 1.5 mV for square-wave currents, mean ± s.e.m., *n* = 7, *p* = 0.086; −1.8 ± 0.6 mV for aEPSGs, *n* = 10, *p* = 0.02). Because the decrease in amplitude was small, and we wished to exclude the potential influence of sodium spikes, Figure 12C and the above statistics were obtained by pooling across conditions that were chosen in each cell to maximize TTD amplitude in the absence of spikes (see Materials and Methods). We conclude that a reduction in T-type calcium current in 1.2 vs. 2.0 mM external calcium was very likely to have contributed to the slower and less complete recovery of excitability at −80 mV (Figures 12A,B).

## Discussion

The present results support the theory of predictive homeostasis (Fiorillo et al., 2014), which proposes that most or all voltage-regulated ion channels in sensory neurons serve a homeostatic role. That T-type calcium channels maintain a homeostatic level of excitability is of particular interest, since unlike many other voltage-regulated channels (e.g., depolarization activated potassium channels, hyperpolarization-activated cation channels), T-type channels are both excitatory and exhibit positive feedback. Our results suggest that T-type channels are specifically designed for restoring excitability following hyperpolarization lasting more than a 100 ms. Such hyperpolarization is likely to be prominent in many neurons that receive opponent-type synaptic inhibition, including ON- and OFF-type neurons throughout the visual system (Ferster, 1988; Hirsch et al., 1998; Wang et al., 2007) as well as midbrain dopamine neurons (Fiorillo, 2013; Fiorillo et al., 2013).

### Experimental evidence

We have provided evidence that T-type calcium channels do not induce a “burst mode” in LGN under natural conditions, but rather work toward maintaining a single optimal and homeostatic I-O relation, as predicted by theory (Dan et al., 1996; Fiorillo et al., 2014). Past evidence for bursts was obtained under conditions in which membrane excitability was very likely to have been unnaturally high. We have identified at least four factors that decrease membrane excitability and counteract the generation of T-type-driven depolarizations under natural conditions, none of which were examined in studies that reported T-type driven bursts with an all-or-none character (Llinás and Jahnsen, 1982; Jahnsen and Llinás, 1984; Zhan et al., 1999; Gutierrez et al., 2001). First, the synaptic conductance of neuronal membranes is much higher in the brain than under typical conditions in brain slices (Destexhe et al., 2003), particularly since both synaptic excitation and inhibition are strong at the time of TTDs. We found that excitability at hyperpolarized potentials nearly matches the homeostatic excitability found at depolarized potentials in the presence (Figures 5, 8) but not absence (Figure 10) of synaptic conductances, consistent with previous evidence that the shunting of membrane current suppresses the all-or-none character of the LTS (Ulrich and Huguenard, 1997a). Second, the standard extracellular concentration of calcium utilized *in vitro* (2.0 mM) is higher than found *in vivo* (1.1–1.5 mM) (Hansen, 1985; Jones and Keep, 1988), and TTDs and membrane excitability at hyperpolarized potentials are reduced at a lower and more physiological concentration of calcium (1.2 mM) (Figure 12). Third, acetylcholine and other modulatory neurotransmitters are typically at higher extracellular concentrations in an intact brain. We found that activation of cholinergic receptors suppresses recovery of membrane excitability following hyperpolarization (Figure 7), consistent with the finding that acetylcholine suppresses the T-type mediated LTS (Zhan et al., 2000).

Fourth, in most studies, including all of those that reported T-type driven bursts with an all-or-none character (Llinás and Jahnsen, 1982; Jahnsen and Llinás, 1984; Zhan et al., 1999; Gutierrez et al., 2001), membrane hyperpolarization was artificially sustained for at least seconds. By contrast, most hyperpolarizations during vision are likely to be brief (less than 250 ms on average) (Wang et al., 2007). Membrane potential is unlikely to reach below −80 mV (the primary membrane potential studied here), since −80 mV is the chloride equilibrium potential (Ulrich and Huguenard, 1997b). Only about 25% of T-type channels are deinactivated after allowing enough time to reach steady state at −80 mV, and fewer are deinactivated at more positive potentials (Coulter et al., 1989; Crunelli et al., 1989). Deinactivation of T-type channels has been found to reach about half of its steady state level after 100 ms (~12%), and may only be complete after 500 ms (based on extrapolation to −80 mV and 37°) (Crunelli et al., 1989; Coulter et al., 1989). Thus, if T-type channels are most often activated following 100–300 ms at −75 to −80 mV, we can infer that the fraction of deinactivated (primed) T-type channels during hyperpolarization may typically be 10–15%. Our results suggest that T-type channels help to restore excitability after such brief hyperpolarization, but that excitability remains substantially below its homeostatic level for at least several 100 more milliseconds (Figures 5–8) (see below for discussion of related theoretical issues).

Previous *in vitro* studies have also provided evidence that T-type channels in TC neurons can contribute to graded amplification of EPSPs, particularly when current is shunted by elevated membrane conductance (Ulrich and Huguenard, 1997a; Timofeev et al., 2001; Wolfart et al., 2005; Wei et al., 2011; Deleuze et al., 2012). Two of these were comparable to the present study in using dynamic clamp to mimic aspects of *in vivo* synaptic conductances (Wolfart et al., 2005; Deleuze et al., 2012). At the end of Materials and Methods we compare our study to these prior studies and we note several respects in which we believe our experimental design to have more closely mimicked natural physiological conditions in the brain, including temporal summation of EPSPs and transient hyperpolarization. Although not motivated by the same theoretical issues, Deleuze et al. (2012) also provided evidence in support of our theory by showing that T-type channels maintain rather than alter I-O relations during prolonged periods of hyperpolarization in somatosensory (ventrobasal) thalamus. The most fundamental advance of the present work over that of Deleuze et al. (2012) is in demonstrating the contribution of T-type channels to maintenance of a precise homeostatic I-O relation that is optimal according to theory (Dan et al., 1996; Fiorillo et al., 2014) and has been found during vision (Dan et al., 1996; Carandini et al., 2007; Sincich et al., 2007; Weyand, 2007; Casti et al., 2008).

Studies performed *in vivo* with intracellular recordings have found that T-type channels contribute to bursts of spikes (Lu et al., 1992; Wang et al., 2007). Although these studies proposed that T-type channels cause the bursts and thereby create a second “burst mode” of firing, I-O relations were not quantified and thus the observed bursts of output spikes may have been driven by bursts of retinal input spikes. The study of Lu et al. (1992) caused hyperpolarization via current injection without mimicking the natural inhibitory synaptic conductance (Lu et al., 1992).

Simultaneous recordings of retinal input and thalamocortical spike output during visual stimulation support the view that retinal input, not T-type conductance, is the proximal cause of spikes in LGN. The vast majority of spikes (>95%) are preceded by a rEPSG within 2 ms, including those within bursts (Sincich et al., 2007); see also (Weyand, 2007). Bursts of spikes following periods (>100 ms) without spikes were always (>99.9%) preceded by “failed” rEPSGs occurring several ms earlier (Sincich et al., 2007). This is consistent with our finding that when T-type channels are primed, a “failed” first rEPSG and TTD are necessary so that a second rEPSG can cause a spike. Likewise, Wang et al. (2007) reported that “many putative T-currents were not large enough to drive the membrane across the threshold for sodium spikes or led to only one sodium spike.” Quantification of the I-O relation with intracellular recordings could provide further evidence, but this has never been done so far as we know. The potential difficulty in counting rEPSGs during high frequency synaptic events could be overcome in future experiments if the timing of rEPSGs is directly controlled by experimenters (rather than occurring at uncertain times in response to a naturalistic movie, as in the study of Wang et al., 2007).

A standard criterion for identification of bursts based on extracellular recordings is an inter-spike interval of 4 ms or less following a period of 100 ms or more with no spikes (Lu et al., 1992; Guido and Weyand, 1995; Reinagel et al., 1999; Weyand et al., 2001; Lesica and Stanley, 2004; Denning and Reinagel, 2005). These bursts have been found to be rare events, contributing less than 1–5% of all spikes in TC neurons of LGN (Guido and Weyand, 1995; Weyand et al., 2001). Our results suggest that these thalamic bursts are caused by bursts of retinal spikes that are amplified by activation of T-type channels, with a homeostatic average of one thalamic spike for two retinal spikes. Given the time required for deinactivation of T-type channels, and the likely prevalence of hyperpolarized periods during vision (feed-forward opponent inhibition should be about as prevalent as retinogeniculate excitation; Wang et al., 2007), our results also suggest that T-type channels will often amplify the effect of rEPSGs, and that this will result in either 0 or 1 spike in the majority of cases (assuming that most retinal firing events consist of either 1 or 2 spikes). Thus, although T-type channels appear not to induce a fundamentally distinct firing mode, they are likely to contribute considerably more to normal visual function than one would expect based on extracellular analyses of burst frequency.

### Relation to theory

We predicted the present results based on a theory that ascribes a homeostatic role to the majority of voltage-sensitive ion channels, and proposes that the properties of such channels are optimized for predicting natural temporal patterns of synaptic input (Fiorillo, 2008; Fiorillo et al., 2014). The remarkable diversity of voltage-regulated ion channels would be needed to maintain homeostasis of membrane excitability given the numerous temporal patterns that are found within the brain and even within a single neuron. Some of these patterns arise from external sensory stimuli whereas others are internally generated (such as the moderately stereotyped shape of a unitary EPSP, as in Figure 1A). If the theory is correct, we should be able to predict the properties of a specific ion channel given knowledge of the statistical patterns of synaptic input and membrane excitability associated with its activation. That T-type channels restore the homeostatic I-O relation without causing bursts implies that the density, voltage dependence, and activation kinetics of the channels are finely tuned to amplify EPSPs toward spike threshold without consistently crossing it. Additional mechanisms may work with T-type channels to help to restore homeostasis following hyperpolarization lasting a 100 ms or more, including depolarization caused by H-type cation channels, and the facilitation of rEPSG amplitude (decay of paired-pulse depression) following long intervals without presynaptic spikes.

If we had greater knowledge of natural statistics we could attempt to predict more specific properties, such as the optimal rate of deinactivation and density of functional (deinactivated) T-type channels. For example, we can imagine the simple scenario that the duration of the hyperpolarized state (prior to a rEPSG) is “random” and thus exponentially distributed with some characteristic and predictable mean. It is naturally better to update predictions and restore homeostasis quickly so as to minimize the period of “blindness,” and thus faster deinactivation would presumably be favored. Alternatively, we can imagine that in addition to the longer-lasting hyperpolarization for which T-type channels are designed, a second pattern of hyperpolarization is brief (<50 ms) and stereotyped without the longer-lasting component (as might happen in the case of a synchronous GABA_A_ IPSP that reaches a peak and immediately decays). In this latter scenario, deinactivation should not be too fast since T-type channels should not contribute to excessive excitability once synaptic inhibition is gone. Thus, we can imagine that the moderate rate of deinactivation of T-type channels could be optimal for channels designed to compensate only for the slower of two common patterns of hyperpolarization. If so, we could say that T-type channels “wait to collect evidence” that the hyperpolarization will be sustained rather than transient before deinactivating.

Despite our lack of detailed knowledge of natural statistical patterns of synaptic input, it is clear that deinactivation of T-type channels must necessarily come at the cost of excessive excitability in some instances (Figure 9). In addition to the informational cost of excessive excitability, there would also be the metabolic cost of T-type calcium currents as well as spikes. These costs would favor lower densities of T-type channels having slower deinactivation. This could account for our observation that excitability was not fully recovered, on average, even after 800 ms at −80 mV. This incomplete recovery of excitability matches the common finding that homeostatic feedback mechanisms typically cause incomplete compensation rather than overcompensation.

The better a neuron's homeostatic mechanisms are able to predict synaptic drive and maintain homeostatic excitability, the more information spikes will carry about sensory stimuli, and the better sensory perception should be. There are many types of visual “contrast adaptation” that are known to improve the ability of neurons and animals to discriminate light intensity (or contrast), and these occur at multiple levels of the visual system through various mechanisms. Our results suggest that T-type channels mediate a specific form of contrast adaptation dedicated to high spatial frequencies (due to the small size of receptive fields in LGN) and temporal frequencies below about 10 Hz (due to the deinactivation rate of T-type channels). We are unaware of any studies that have looked for this particular sort of contrast adaptation, which might be revealed in psychophysical experiments by use of a high-contrast checkerboard pattern as an “adapting” stimulus followed by low-contrast “test” increments of opposite polarity. For example, the high-contrast adapting stimulus should hyperpolarize a set of ON-type thalamocortical neurons that have “dark” in the center of their receptive fields. If a low-contrast test increment is subsequently made in the light intensity (the darkness in the receptive field center of these ON-type cells suddenly becomes less dark), this would be expected to cause synaptic excitation of those ON-type neurons. If this test increment occurs 50 ms after onset of the adapting stimulus (with no gap between offset of adapting stimulus and onset of test stimulus), when T-type channels are inactivated, the ON-type neurons should have synaptic excitation but no spikes, and psychophysical tests should show low sensitivity to the increments. When the same test increments are delivered instead after a few hundred ms of the high-contrast stimulus, when T-type channels have had time to deinactivate, the test increments should evoke spikes (in roughly half the trials and in half the cells), and perceptual discrimination of the increment intensity should be improved relative to the case of shorter adaptation times. Furthermore, theory would predict that psychophysical sensitivity to luminance would be greatest in response to the specific test increment that evokes spikes in half the trials and in half the relevant cells (ON-type cells with appropriate spatial receptive fields in this example) (Fiorillo et al., 2014). Nonetheless, at any time during which the relevant neurons are substantially hyperpolarized from rest (about −65 mV), we would expect perceptual sensitivity to be inferior to the more prevalent situation in which neurons are closer to resting potential, and predictions are highly accurate in the presence of static and low-contrast visual stimulation (low spatial and temporal frequencies).

Principles of information and prediction have been highly successful in explaining properties of early sensory systems in relation to natural patterns of external sensory stimuli (Simoncelli and Olshausen, 2001). Application of related theoretical principles at the cellular level could allow us to explain the diversity of ion channel properties and their informational content in relation to natural patterns of local synaptic input (Fiorillo, 2008, 2012; Fiorillo et al., 2014). In addition to providing experimental evidence supporting the theory, the present work provides the first specific and detailed illustration of how the theory can be tested.

## Author contributions

Su Z. Hong and Christopher D. Fiorillo designed the experiments. Su Z. Hong and Haram R. Kim performed the experiments. Christopher D. Fiorillo and Su Z. Hong wrote the manuscript.

### Conflict of interest statement

The authors declare that the research was conducted in the absence of any commercial or financial relationships that could be construed as a potential conflict of interest.
